# Circulating Microclots Are Structurally Associated With Neutrophil Extracellular Traps and Their Amounts Are Elevated in Long COVID Patients

**DOI:** 10.1002/jmv.70613

**Published:** 2025-10-02

**Authors:** Alain R. Thierry, Tom Usher, Cynthia Sanchez, Simone Turner, Chantelle Venter, Brice Pastor, Maxine Waters, Anel Thompson, Alexia Mirandola, Ekaterina Pisareva, Corinne Prevostel, Gert J. Laubscher, Douglas B. Kell, Etheresia Pretorius

**Affiliations:** ^1^ IRCM, Montpellier Cancer Research Institute, INSERM U1194 Montpellier University Montpellier France; ^2^ ICM, Institut Régional du Cancer de Montpellier Montpellier France; ^3^ Department of Physiological Sciences, Faculty of Science Stellenbosch University, Stellenbosch Private Bag X1 Matieland Stellenbosch South Africa; ^4^ Mediclinic Stellenbosch, Stellenbosch Stellenbosch South Africa; ^5^ Department of Biochemistry, Cell and Systems Biology, Institute of Systems, Molecular and Integrative Biology, Faculty of Health and Life Sciences University of Liverpool Liverpool UK; ^6^ The Novo Nordisk Foundation Centre for Biosustainability Technical University of Denmark Kongens Denmark

**Keywords:** circulating DNA, immunothrombosis, Long COVID, microclots, myeloperoxidase, neutrophil elastase, neutrophil extracellular traps

## Abstract

The persistence of vasculo‐thrombotic complications has been put forward as a possible contributing factor in the Long COVID (LC) syndrome. Given the recently reported separate demonstration of the association of LC with elevated levels of heterogenous fibrin(ogen) amyloidogenic particles (microclots) and with those neutrophil extracellular traps (NETs), markers that are linked to thromboinflammation, this study considers the association of microclots with NETs. The results show that NETs markers (Myeloperoxydase, Neutrophil Elastase, and circulating DNA) are quantitatively and structurally associated with the size and number of microclots in patients with LC. These markers showed a strong diagnostic performance, both independently and when combined. Our study revealed that NETs may be a component of circulating microclots. We suggest that higher NETs formation might promote the stabilization of microclots in the circulation, potentially leading to deleterious effects which contribute causally to the LC syndrome.

## 1 Introduction

In the aftermath of the global SARS‐CoV‐2 pandemic, Long COVID (LC) has been recognized as a widespread and challenging condition, with symptoms that impair multiple organ systems and affect millions worldwide. Current estimates indicate that approximately 10%–30% of individuals infected with SARS‐CoV‐2 will experience some form of LC, underscoring the urgent need for effective diagnostic markers and a deeper understanding of its pathophysiology [1, 2, 3]. Amidst this backdrop, our research details the significant association and potential mechanistic link between microclots [4, 5] and neutrophil extracellular traps (NETs) [6, 7]. These findings not only suggest novel diagnostic pathways but also deepen our understanding of the thromboinflammatory processes and autoimmunity underlying LC [8]. By exploring the synergistic and potentially orthogonal relationships between microclots and NET markers, we argue for their combined diagnostic potential, paving the way for more targeted and personalized management strategies to confront this enduring health issue.

Increased risks of thrombotic complications such as pulmonary embolism and deep vein thrombosis have been reported more than 6 months after SARS‐CoV2 infection, particularly in patients who have previously suffered severe illness [2, 3]. This suggests the manner in which hypercoagulation how hypercoagulation may contribute to the varied symptoms experienced by patients with LC, through mechanisms such as capillary blockage, chronic ischaemia‐reperfusion injury, and autoantibody formation. Generally speaking, the state of hypercoagulation can affect multiple organs. Although it is known that LC is associated with factors leading to immune dysregulation and tissue damage [1, 2, 3], the aetiological relationship between LC and coagulation is still under investigation by various groups.

The mechanism of healthy blood clotting, culminating in the self‐assembly of soluble fibrinogen molecules cleaved by thrombin to make insoluble fibrin is well‐understood [9]. The resulting fibrin mesh is made up of long fibers, typically with a diameter in the 50–100 nm range. As early as 2008 we suggested that fibrin ultrastructural changes could be useful in identifying disease patterns [9], and referred to the anomalous clots observed as ‘dense matted deposits’ [9]. We also found that these dense matted deposits were resistant to fibrinolysis, and that this anomalous form could be stained with amyloid stains such as Thioflavin T (ThT) [4]. Whereas our initial focus was on the study of clotting pathology in the presence of thrombin, over the years this has evolved, ultimately leading to the discovery that direct protein‐protein interactions between circulating inflammatory molecules and soluble fibrinogen are the main drivers of clotting pathologies. The discovery of circulating amyloid fibrin(ogen) marked a crucial milestone, not least due to its significant prevalence in acute COVID‐19 cases [5]. This shifted the focus to the biochemical and structural characteristics of fibrin(ogen), highlighting a conformational change into an amyloidogenic structure triggered by the presence of circulating inflammatory molecules. These initial discoveries illuminated how such molecules could directly influence pathological clotting, as well as erythrocyte and platelet pathology.

We also showed that these fibrinolytic‐resistant microclots entrap various inflammatory molecules, including proteins that prevent clot breakdown (alpha‐2‐antiplasmin, e.g.) [10, 11], and that the extent of the microclots, with their trapped inflammatory molecules, is a key influencer in LC pathology. We also showed that the simple presence of the spike protein S1 from SARS‐CoV‐2 is sufficient to induce fibrinolytic ‐resistant microclots. Recognising that a reliable, quantitative method was needed for measuring microclots in platelet‐poor plasma, we developed a cell‐free imaging flow cytometer method for the detection and quantification of microclots [12]. Microscopy methods are also effective in the analysis of such microclots, as observed in sepsis [13].

Although fibrinogen is a central part and scaffold of some of the microclots, the ThT binds to any amyloidogenic and misfolded protein associated/incorporated into the microclot. It is likely that most of the dysregulated inflammatory molecules we found inside the microclots have a significant amyloidogenicity themselves. While the microclots have a diverse/varying content, the term “microclots” was largely used in the litterature. In this study, we use the term “microclots” to refer to small, circulating fibrin amyloidogenic complexes that may form independently of thrombin and are resistant to fibrinolysis.

NETs [6, 7, 14] have also been found to be associated with COVID [15, 16, 17, 18, 19, 20] and LC [21], and have long been associated with inflammatory diseases [22]. NETs formation (NETosis) is produced by activated neutrophils and is part of the innate immune response. NETs are composed of chromatin and of several proteins, derived notably from granules (e.g. Neutrophil elastase, Myeloperoxidase, bactericidal peptides, cathepsin G, lactoferrin and matrix metalloproteinase‐9), each contributing to the elimination of bacteria [22, 23]. This allows NETs to eliminate microbes within the first hours of infection, doing so physically by means of the DNA fibers, and chemically by means of potent enzymes (NE and MPO, for instance). However, uncontrolled and excessive NETs formation may be deleterious, and has been associated with various sterile and non‐sterile disorders, called NETopathies [23, 24, 25]. These are mainly inflammatory, and include microbial infection, autoimmune diseases, cancer, diabetes, arthritis, and others [17, 22]. DNA is the main NETs component, and the principal NETs byproducts consist of specifically degraded or fragmented DNA that are shed into the bloodstream (circulating DNA, cirDNA) [26]. Given that NETs formation has been found to be associated with cirDNA in various inflammatory diseases [22, 27, 28], and that the production of cirDNA was recently shown to derive directly from NETs degradation [26], cirDNA may represent a biomarker of NETs formation. The participation of NETs in thrombus formation has previously been suggested, based on the observation of thrombotic events in various NETopathies [17, 22, 29]. In addition, the *ex vivo* release of intertwined fibrin and NETs scaffolds by isolated, stimulated neutrophils in fibrin clots has been observed. For instance, NETs markers have been detected in a coronary thrombus removed with percutaneous coronary thromboaspiration from a patient with acute myocardial infarction [30].

## 2 Materials and Methods

### 2.1 Objectives and Study Design

We have separately revealed both the presence of microclots [1, 4, 7], and elevated cirDNA and NETs in LC, and we here assess the extent to which they are linked/associated. We used the same cohort of healthy individuals (HI) and individuals with LC (HI, Table S1 and LC, Table S2) show the demographics and co‐morbidities of LC and both HI cohorts). We first determined the number of microclots and their size, and simultaneously determined the plasma concentrations of NETs protein markers (MPO, NE) and total cirDNA, and performed correlation tests. We then assessed whether NETs constituents were present within the microclots, seeking to determine a possible structural association between the two entities.

### 2.2 Study Cohort and Sample Demographics

We included two control cohorts and one Long COVID cohort. The control cohorts were as follows: one from South Africa [SA controls, (*n* = 14)] and another from France [EFS controls, (*n* = 24)], both comprising healthy individuals without LC symptoms at blood collection. The inclusion of these geographically and culturally distinct control groups aimed to explore any differences or similarities in inflammatory profiles. The South African control cohort included 14 healthy individuals [seven males, seven females; mean age 28 (26–42)] with no cardiovascular history, coagulation disorders, or pregnancy. Two participants had hypercholesterolemia, and one had hypertension, all effectively managed through diet and treatment. The French control cohort consisted of 24 healthy blood donors [15 males, nine females; median age 45.5 (19–64)] with similar exclusion criteria. Demographic details and co‐morbidities were documented and compared across cohorts.

For the LC group, 50 patients [18 males, 32 females; mean age 50 ( ± 17)] were recruited through clinical collaborators who identified post‐acute COVID‐19 individuals with persistent LC symptoms. LC patients completed a questionnaire detailing their demographics, comorbidities, medication use, COVID‐19 vaccination history, severity of initial infection, and self‐reported LC symptoms. All participants provided informed consent, and ethical clearance was secured before blood collection. Platelet‐poor plasma samples were cold‐shipped between South Africa and France for analysis. Individuals constituting both HI cohorts (EFS and SA HI), included in this study were selected to not be on any anticoagulant therapy, baby aspirin or on any other medication that might impact their clotting physiology.

### 2.3 Ethical Clearance

The investigation received ethical approval from the Health Research Ethics Committee (HREC) at Stellenbosch University in South Africa, under the references: N19/03/043 and project ID: 9521 with annual re‐approval. The French ethics approval number is EFS‐PM N° 21PLER2018‐0069. We have a data and material sharing agreement between Stellenbosch University and INSERM (S008426). Participants were recruited based on the information they provided to their clinicians in collaboration with the study. Before sample collection, participants were extensively briefed on the study's objectives, potential risks, and details of the entire process to gain their informed consent. Throughout all study procedures there was mandatory adherence to ethical guidelines and principles, as detailed by the Declaration of Helsinki, South African Guidelines for Good Clinical Practice, and Medical Research Council Ethical Guidelines for Research.

### 2.4 Blood Sample Collection and Preparation

Blood samples from South Africa was collected in citrate tubes, either used fresh to study blood cells or platelet‐poor plasma was created and stored at −80°C. A portion of the frozen PPP was cold shipped to the INSERM laboratory in France. Blood samples form the French cohort were obtained from the Etablissement Français du Sang (EFS), which is Montpellier's blood transfusion center (Convention EFS‐PM N° 21PLER2018‐0069). Blood sample from healthy individuals was collected in 5‐mL Streck tubes. No difference in terms of NETs markers and cirDNA was found when comparing EDTA vs Streck tubes. As previously determined, no bias from EDTA was observed in the quantified markers in our study [17, 22, 27, 29].

### 2.5 Fluorescence Microscopy of Platelets Remaining in the Blood Cell Fraction After Centrifugation and Removal of Platelet‐Poor Plasma

Platelets from the South African controls and LC samples were studied in the cellular fraction that remained after whole blood was centrifuged at 3000×g for 15 min. CD62PE (P‐selectin on platelet surface) (IM1759U, Beckman Coulter, Brea, CA, USA) and to PAC‐1 (activated glycoprotein (GP) IIb/IIIa) (340507, BD Biosciences, San Jose, CA, USA) was added to the hematocrit. P‐selectin is released from the cellular granules during platelet activation and then moves to the surface of the platelet membrane. The antibody PAC‐1 detects the neoepitope of active GPIIb/IIIa. PAC‐1 antibody binding is correlated with platelet activation. After the samples was incubated at room temperature for 30 min, samples were also viewed using the Zeiss Axio Observer 7 fluorescence microscope with a Plan‐Apochromat 63×/1.4 Oil DIC M27 objective (Carl Zeiss Microscopy, Munich, Germany). We used excitation wavelength 450 to 488 nm and the emission at 499 to 529 nm (PAC‐1), and excitation 540 to 570 nm and the emission 577 to 607 nm (CD62PE).

### 2.6 Whole Blood Analysis (Platelets, Red Blood Cells and White Blood Cells) Using Imaging Flow Cytometry

For imaging flow cytometry (using various fractions of the sample to anayse the blood cells), we used the Amnis® FlowSight® Imaging Flow Cytometer (Luminex, Seattle, WA, USA). This method integrates fluorescence microscopy for single‐cell morphology analysis with the capabilities of imaging flow cytometry, enabling precise discrimination of heterogenous cell populations and high flow rate capacity while maintaining data quality.

#### 2.6.1 Platelets and Red Blood Cell Imaging Flow Cytometer Analysis Using Whole Blood

To study intact platelets and red blood cells in whole blood, 10 µL of the whole blood was added to a blood preparation tube, followed by the addition of where 4 µL CD62P (PE‐conjugated), 4 µL PAC‐1 (FITC‐conjugated) and 1 µL CD235a (349132, BioLegend, San Diego, CA, USA). The samples were incubated for 30 min, at room temperature (protected from light). After the incubation step, the whole blood was diluted (1:1000) in PBS, followed by analysed with the Amnis® FlowSight® Imaging Flow Cytometer, using the 405 and 488 nm lasers. The PAC‐1 signal were viewed in Channel 2, CD62 signal were viewed in Channel 3 using the 488 nm laser and the CD235a signal were viewed in Channel 7 using the 405 nm laser. All samples were acquired using the same acquisition template for 5 min each and then the data was analysed with IDEAS 6.2 software.

#### 2.6.2 White Blood Cell Imaging Flow Cytometer Analysis Using the Buffy Coat

To study white blood cells using the imaging flow cytometer, the buffy coat was used. This buffy coat layer contains the white blood cells after whole blood is centrifuged and platelet‐poor plasma, removed. 50 µL buffy coat was exposed to 5 μL CD45‐PC5 (IM2652U, Beckman Coulter, Brea, CA, USA) and 50 μL of Hoechst 33342 (20 mM) (Thermo Fisher Scientific, Waltham, USA). After the incubation step (at room temperature for 30 min), the buffy coat was diluted (1:1000) in PBS, followed by analysis with the imaing flow cytometer. A template was used, utilising the 405 and 488 nm lasers visualized in channel 5 (CD45) and channel 7 (Hoechst). Sample acquisition was conducted over a 5 min duration, whereafter the data was analysed with IDEAS 6.2 software.

### 2.7 Microscopy and Imaging Flow Cytometry Analysis of Platelet‐Poor Plasma

Microscopy and imaging flow cytometry analyses were conducted on plasma prepared by 3000×g centrifugation for 15 min in LC patients, and by a two‐step centrifugation process (1200 g for 10 min followed by 16000 g for 10 min) in the in both HI SA and EFS control cohorts. As shown in the Supporting Information, both processes yielded similar values and observations for the tested markers, including microscopy, microclots counts, MPO, NE, and cirDNA.

#### 2.7.1 Fluorescence Microscopy of Microclots in Platelet‐Poor Plasma Samples

For this analysis, platelet‐poor plasma (PPP) was used from all the 3 cohorts (controls and LC samples from South Africa and controls from France). The PPP was created by a centrifugation step of 3000×g for 15 min. It is well‐known that platelet‐poor plasma that are devoid of intact cells, is created using this centrifugation step and spinning time [31]. The PPP was stored in 1.5 mL Eppendorf tubes, and then frozen at −80°C until analysis.

### 2.8 Analysis and Quantification of cirDNA, Myeloperoxidase and Neutrophil Elastase Assay

Determination of cirDNA concentration and NETs protein markers (NE and MPO) were performed from exactly the same plasma sample as prepared following a two steps centrifugation process (1200 g for 10 min then 16000 g for 10 min). This preparation was previously chosen to exclude large vesicles that may encapsulate DNA and consequently, contaminate the fraction that should only contains circulating cell‐free DNA (cirDNA). We previously demonstrated the absence of cells and cell debris in published guidelines as well as to set clinical studies towards evaluating cirDNA diagnostic performance in cancer and COVID/Long COVID patients [17, 22, 29, 32]. Additionally, in several reports, we showed that the combination of the three analytes provides an accurate measure of NETosis [27, 29].

#### 2.8.1 Plasma Isolation and cirDNA Extraction

Blood sample from healthy individuals was centrifuged at 1200 g at 4°C for 10 min, and the plasma supernatants were immediately centrifuged at 16,000 g at 4°C for 10 min. Afterward, the plasma was either immediately used for DNA extraction or stored at −20°C. CirDNA was extracted from 50 µL of plasma using the Maxwell® RSC Blood DNA Kit (Promega) according to the “RSC ccfDNA plasma” in an elution volume of 100 µL. Until they were used, DNA extracts were stored at −20°C. The same extraction procedure was used for the LC‐19 patients and group control.

#### 2.8.2 Quantification of cirDNA

Quantitative PCR IntPlex® method was employed to analyze cirDNA following MIQE recommendations (Bustin, 2010; Bustin et al., 2009), as previously described (Mouliere et al., 2014; Thierry et al., 2014). Total cirDNA concentration in each sample was measured using amplification of a 67 bp wild‐type KRAS gene sequence (amplification primers were 5′CCTTGGGTTTCAAGTTATATG3′ sense and 3′CCCTGACATACTCCCAAGGA5′ antisense). Q‐PCR was conducted in a 25 μl reaction volume on a CFX96 instrument with CFX management software 3.0 (Bio‐Rad). The PCR reaction mixture included Bio‐Rad Super mix SYBR Green PCR mix, amplification primers, PCR‐analyzed water, and DNA extract. Thermal cycling involved a 3‐min Hot‐start Polymerase activation denaturation step at 95°C, followed by 40 cycles at 95°C for 10 s and 60°C for 30 s. Melting curves were obtained from 55°C to 90°C with a plate reading every 0.2°C. Quantifications were calibrated using serial dilutions of DIFI cell line genomic DNA, and sample concentrations were extrapolated using the standard curve. All experiments included duplicate samples, and DNA quantification data were validated using an internal control with a known concentration.

#### 2.8.3 Myeloperoxidase and Neutrophil Elastase Assay

MPO and NE concentrations were determined using ELISA according to the manufacturer's instructions (Duoset R&D Systems, DY008, DY3174, and DY9167‐05),. Captured antibodies were diluted with Reagent Diluent (RD) from Supporting reagent kits (DY008), coated on 96‐well microplates, and incubated overnight at room temperature. After clearing and washing with Wash Buffer (WB), microplates were blocked with RD for 2 h. After washing, 100 µL of diluted 1/10 plasma samples, standards, and controls were added and incubated for 1 h at room temperature. The standard curve ranged from 0.12 to 8 ng/mL. After removal and washing, detection antibodies diluted in RD were added for 1 h. Following another wash, microplates were incubated for 30 min at room temperature with streptavidin‐HRP. After three washes, substrate solution (100 µL per well) was added, and after a 10‐min incubation, the PHERAstar FS instrument, in conjunction with the PHERAstar control software, promptly read the Optical Density (O.D) at 450 nm for each well.

#### 2.8.4 Fluorescense Microscopy of Purified Fibrinogen and Platelet‐Poor Plasma

We optimized a fluorescence microscopy assay to confirm the presence of fibrin(ogen) in microclots. A 4 mg/mL solution of purified soluble fibrinogen (Thermo‐Fisher, Rp43142) was treated with spike protein (final exposure concentration 100 ng/mL) or lipopolysaccharide (final exposure concentration LPS, 50 ng/L), followed by staining with Fibrinogen α‐chain antibody (Alexa Fluor 594, ab216367) and Thioflavin T (ThT). This demonstrated that spike protein or LPS addition induce the formation of insoluble microclots, with fibrin(ogen) binding marked by the α‐chain antibody and amyloid regions highlighted by ThT. To confirm assay reliability, we further tested both healthy and Long COVID samples using MPO (48‐1299‐42, Invitrogen, Waltham, MA, USA), Fibrinogen α‐chain antibody and ThT staining in various combinations using confocal microscopy (Zeiss, LSM 780 confocal microscope).

For the confocal analysis, PPP samples were exposed to MPO, ThT and fibrinogen α‐chain antibody for 30 min (protected from light). A 5 µL drop of each of the samples were placed on microscope slides. The sample drop was allowed to dry for about 5 min. Thereafter it was viewed with the Zeiss LSM780 confocal microscope with a Plan Apochromat 63x/1.4 Oil DIC M27 objective (Carl Zeiss Microscopy, Munich, Germany). The different antibodies were excited by the 405 nm laser (MPO), 488 nm laser (ThT) and 561 nm laser (fibrinogen α‐chain antibody). When microclots were detected a z‐stack was taken (see Suppl Figure S6).

### 2.9 AI and Machine Learning Assistance

Plasma marker data were analyzed using a supervised machine learning framework implemented in R. All features were normalized using Z‐score scaling (mean‐centering and standard deviation scaling) with the preProcess function from the caret package. A Random Forest classifier was trained using the train() function (method = “rf”), applying five‐fold cross‐validation repeated 100 times (repeatedcv) to ensure robust model evaluation. Class probabilities were enabled, and model performance was assessed using the area under the receiver operating characteristic curve (AUC) via the twoClassSummary metric.

Model predictions were used to compute AUC, sensitivity, and specificity at the optimal ROC point (determined as the maximum distance from the diagonal), and confidence intervals were estimated by bootstrapping with 1,000 iterations using the boot and pROC packages. Accuracy and its 95% confidence interval were also calculated using bootstrap resampling. A confusion matrix was generated and visualized as a heatmap showing classification performance as percentages. Reported confusion matrices represent the average of matrices obtained on the test sets of 100 repeated fivefold CV. In addition to the Random Forest model, k‐nearest neighbors (kNN) and Decision Tree models were also evaluated using the same cross‐validation setup, normalization procedure, performance metrics, and bootstrap‐based statistical analysis. This allowed for consistent comparison across classifier types. Feature importance scores from the Random Forest model were extracted using *varImp()* and visualized in a ranked bar plot of the top 20 contributing protein markers.

All analyses were performed in R (version 4.3.3), with random seed (123) initialization for reproducibility and the software packages: *randomForest* (v 4.7‐1.2) for RF models, *caret* (v 7.0‐1) for cross‐validation and confusion matrix extraction, *pROC* (v 1.18.5) for AUC calculation, *ggplot2* (3.5.1) for plotting, *boot* (1.3‐31) for bootstrapping.

To enhance the clarity and detail of the fluorescence microscopy image, we employed a two‐step process utilizing Photoshop and Topaz Gigapixel AI.

### 2.10 Statistical Analysis

The choice of statistical tests was made based on the nature of the data and the specific objectives of the analysis. For comparing groups with non‐parametric data, the Mann‐Whitney U test was selected. This test is appropriate when the data deviate from a Gaussian distribution, as confirmed by the Shapiro‐Wilk test.

For exploring relationships between variables, correlation analysis was conducted using the Spearman test. The Spearman test evaluates the strength and direction of association between variables without assuming linearity. It is particularly appropriate when the relationship between variables may be monotonic but not necessarily linear, or when outliers are present in the data. This test calculates the Spearman correlation coefficient, which ranges from −1 to 1, with values closer to 1 indicating a strong positive correlation, values closer to −1 indicating a strong negative correlation, and values around 0 indicating no correlation. Significant difference between two groups using Spearman correlation matrices are called when the p value shows statistic difference; the R value being indicative.

All statistical analyses were performed using Graph Pad Prism 8.3.1 software. A significance level of 0.05 was chosen to determine statistical significance, meaning that results with a *p*‐value less than 0.05 were considered statistically significant. To provide a clear indication of the level of significance, *p*‐values were reported with asterisk notation: *p < 0.05, **p < 0.01, ***p < 0.001, and ****p < 0.0001.

## 3 Results and Discussion

**Figure 1:**
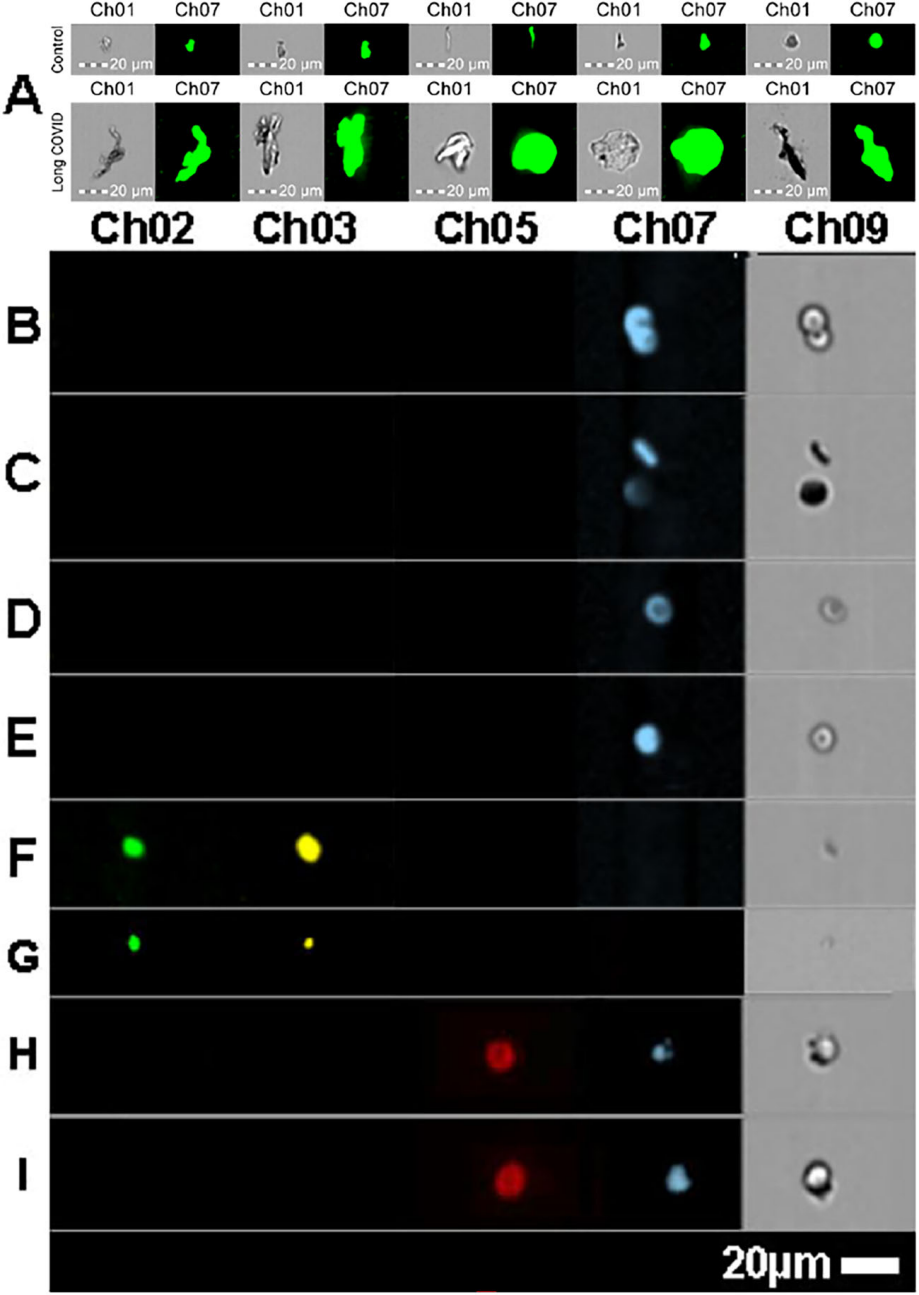
Comparison of the various blood cells and microclots as determined by flow cytometry: A: microclots (microclots) stained with ThT (Ch07) of healthy and Long COVID patients indicating the different sizes and shapes. B‐E: Red blood cells stained with CD235a antibody (Ch07), where the typical biconcave red blood cell morphology can be seen. F and G: Platelets stained with PAC‐1 (CH02) and CD62PE (Ch03), where F is an activated platelet and G is a typical platelet, not showing activation. H and I**:** White blood cells stained with CD45 (Ch05) and Hoechst (CH07). For all the images the bright flied images are in CH09.

Figure 1 shows microclots in platelet‐poor plasma (Figure 1A), and also white blood cells, red blood cells and platelets in whole blood (Figure 1B–I). We used red blood cell, white blood cell, and platelet‐specific antibodies to analyze these cell types in whole blood via imaging flow cytometry. This approach allowed us to clearly differentiate the structure and sizes of these cells in whole blood from the microclots observed in platelet‐poor plasma. Additionally, when these cell‐specific markers were applied to platelet‐poor plasma, no red blood cells, white blood cells, or platelets were detected, confirming the absence of these cell types in the plasma samples. (See further discussion about preparation methods in S10).

### 3.1 Circulating Microclots and NETs Markers Quantitative Association

#### 3.1.1 Elevated Microclots Count in LC Patients

**Figure 2:**
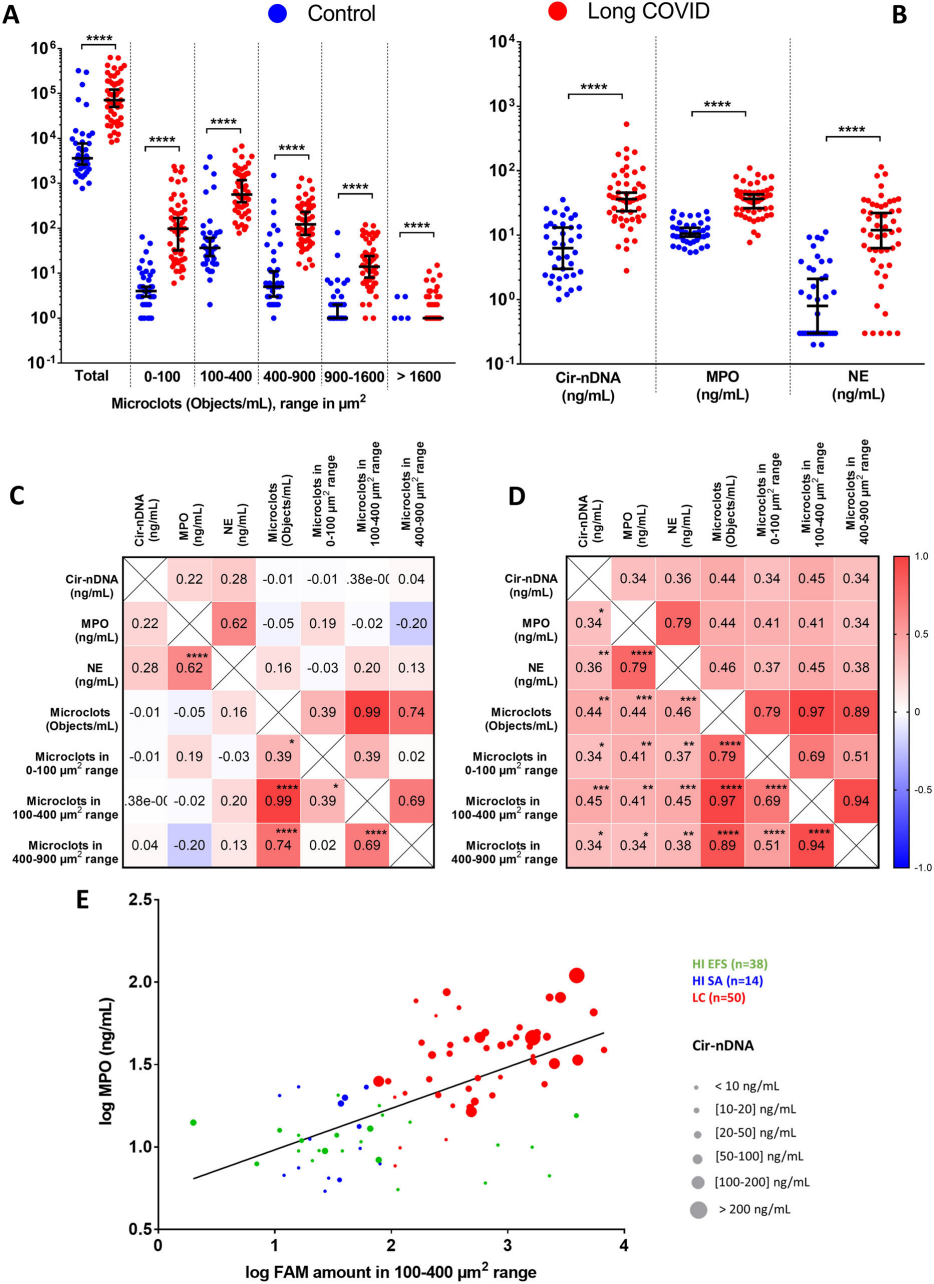
Comparison of Healthy Individuals (HI) and LC plasma patients (LC) across various parameters: (A) Number of microclots (microclots) per milliliter (objects/mL) upon increasing size ranges from 0 to 100 um^2^ to 1600 + µm^2^ as determined by flow cytometry; (B) Concentrations of Cir‐nDNA, MPO, and NE (ng/mL). Median with 95% confidence intervals are represented by lines. Mann‐Whitney tests were employed to compare these parameters between the two groups. A probability of less than 0.05 was considered statistically significant; *p < 0.05; **p < 0.01; ****p < 0.0001. Each dot represents an individual value. (C and D) Correlation matrix of the ir‐nDNA, MPO, and NE concentrations (ng/mL) and the total number of microclots (objects/milliliter) determined in (C) HI (*n* = 38) and (D) LC patients (*n* = 50) plasma. The heatmap illustrates the strength of relationship by Spearman's correlation analysis (red: positive correlation; blue: negative correlation). (E) Scatter plot presentation of values of MPO concentration and microclots amount in 100–400 um^2^ range. Each dot represents an individual. Line represents regression analysis. As shown in Fig.S3 and Table S3–5, there is no statistical difference in markers values when comparing two independent HI cohorts from South Africa (SA HI) and French (EFS HI) populations. SA and EFS HI composed studied HI cohort. Green, EFS HI; blue, SA HI; and red, LC patients. A probability of less than 0.05 was considered statistically significant; *p < 0.05; **p < 0.01; ****p < 0.0001. Cir‐nDNA, circulating nuclear cell‐free DNA; MPO, myeloperoxidase; NE, neutrophil elastase.

We previously developed an imaging flow cytometry method to quantify microclots in platelet‐poor plasma [12]. As determined by imaging flow cytometry, the total number of microclots is statistically much higher in patients with LC than in healthy individuals (P = 1.97 × 10^−12^, 19.7‐fold median difference; Figure 1A and B, Table S3–11). For all arbitrarily selected microclot size ranges ( < 100, 100–400, 400–900, 900–1600, and > 1600 µm²), the median number of microclots is statistically and significantly higher in patients with LC than in healthy individuals (Figure 2A–C). Note, the number of microclots is greatest within the 100–400 µm² size range and lowest in the size fraction > 1600 µm² (Figure 2A). This is in accordance with our previous results on controls and LC [12]. All patients with LC and almost all HI have some microclots of up to 30 µm (Table S3). In contrast, it is notable that 68.4% and 13.1% of healthy individuals have in their plasma microclots in the 900–1600 µm² range and > 1600 µm², respectively (Table S11). In individuals with LC, the proportion of patients showing microclots in these two size ranges is much higher: 98% and 60%, respectively (1.4‐ and 4.6‐fold greater, respectively; Table S11), indicating that the microclots may be greater in size as well as in numbers in LC patients. The imaging flow cytometry results presented here reveal both a significant microclot presence and an increase in both numbers and sizes in the LC samples (Figures 1A and B and 2A and suppl Figure S1A,B). This is in line with previous results [12].

#### 3.1.2 NETs Markers and Microclots Counts are Quantitatively Associated and Discriminate LC From HI Individuals

The plasma concentrations of circulating DNA, MPO and NE are statistically much higher in patients with LC than in healthy patients (5.7‐, 3.5‐ and 14.9‐fold; Figure 2B and Fig. S2 and S3). This confirms our previously published results [21]. The Spearman analysis‐based correlation study shows that in healthy individuals only the concentrations of NE and MPO are statistically associated, as has been demonstrated previously [16, 21]. On the other hand, the concentrations of NE, MPO, and cirDNA, as well as the total number of microclots, are all clearly associated in the plasma of individuals with LC (Figure 2C,D). The number in the 100–400 µm² size range showed the highest discrimination between LC and HI and the highest correlation value with cirDNA, MPO and NE (Figure 2C and D). The number of microclots appears to be especially discriminating between LC and HI: a comparison of the cohorts of healthy individuals and individuals with LC shows the area under the ROC curves to be 0.90; 0.95; 0.90; 0.89; 0.88 and 0.74 for the total number of microclots, the number determined within size ranges of 0–100, 100–400, 400–900, 900–1600 and > 1600 µm², respectively (Figure S4). Furthermore, the concentrations of cirDNA, MPO, NE and the three combined markers are also very discriminating (AUROC 0.90; 0.94; 0.85 and 0.95; respectively; Figure S5), confirming our previous results [21]. When scatter plotting the markers with the highest AUROC (MPO and the 100‐400 µm² size range microclots count), HI and LC patients are distinguishable (Figure 2E).

### 3.2 Structural Association of NETs With Circulating Microclots

**Figure 3:**
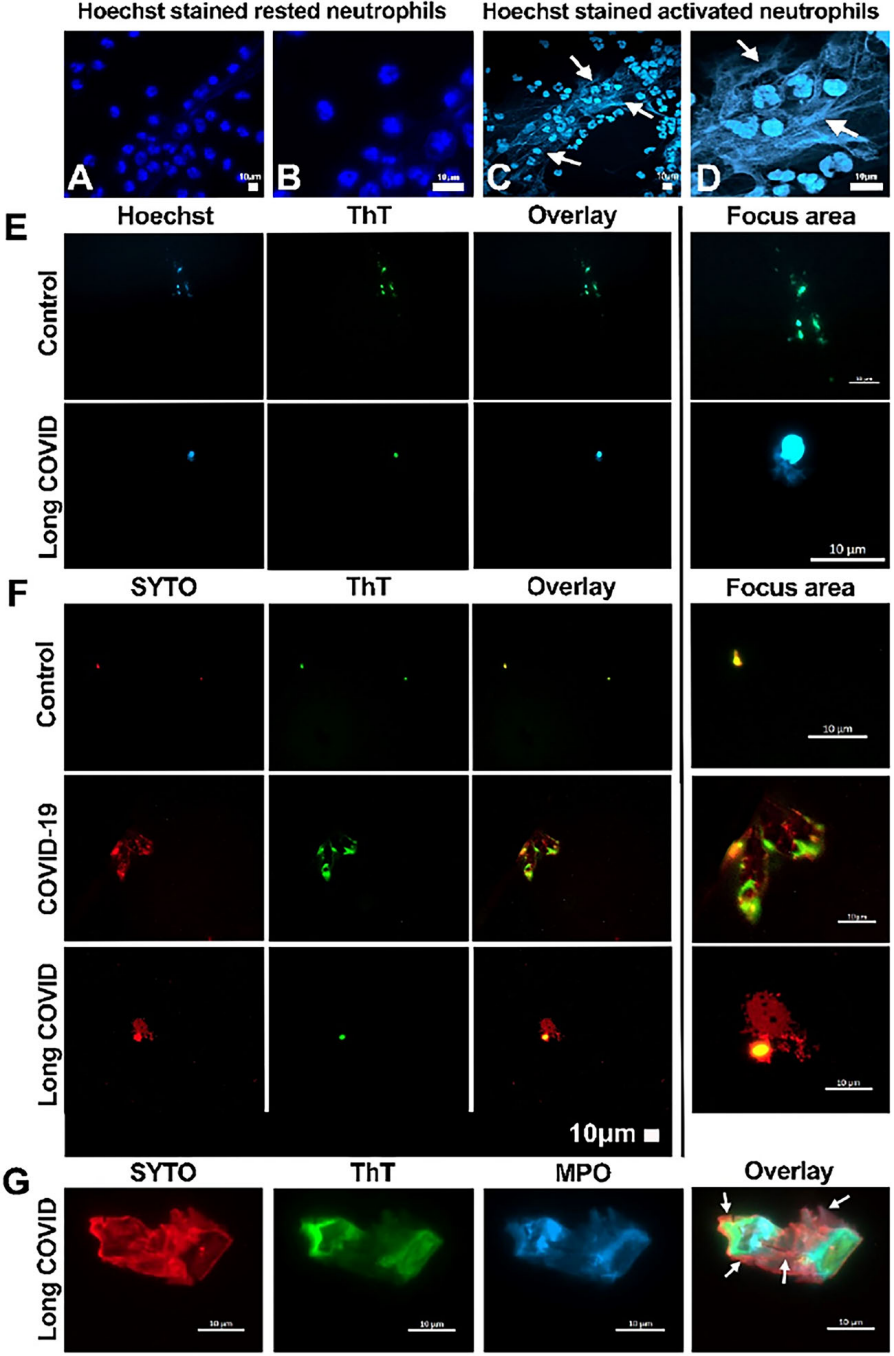
Fluorescence micrographs showing examples of the formation of NETs from resting (A and B) and activated (C and D) neutrophils. Activation of “*ex vivo*” cultured isolated human neutrophils was performed by using PMA treatment for 6 h. Also shown are micrograph examples of microclots detected with Thioflavin T (ThT) and NETs detection using Hoechst stain or SYTO stain of different inflammatory diseases, including acute COVID‐19 and LC compared to the control. The first two columns indicate the Hoechst (blue) or SYTO (red) and ThT (green) stains separately, with the overlay in column three. The area of interest has been enhanced in the last column (E and F). The last row of micrographs (G) are examples of PPP showing myeloperoxidase (MPO) (blue) and SYTO (DNA ‐ red) captured in the microclot (ThT ‐ green) (arrows indicate specific localization of the DNA showing NETs surrounding the microclot, NETs outside the microclot (binding to the apparent two microclots) or (between the apparent two microclots). Experimental conditions for presented here is exposing stored platelet‐poor plasma (PPP) to Thioflavin T and Hoechst or SYTO and MPO to detect microclots.

**Figure 4:**
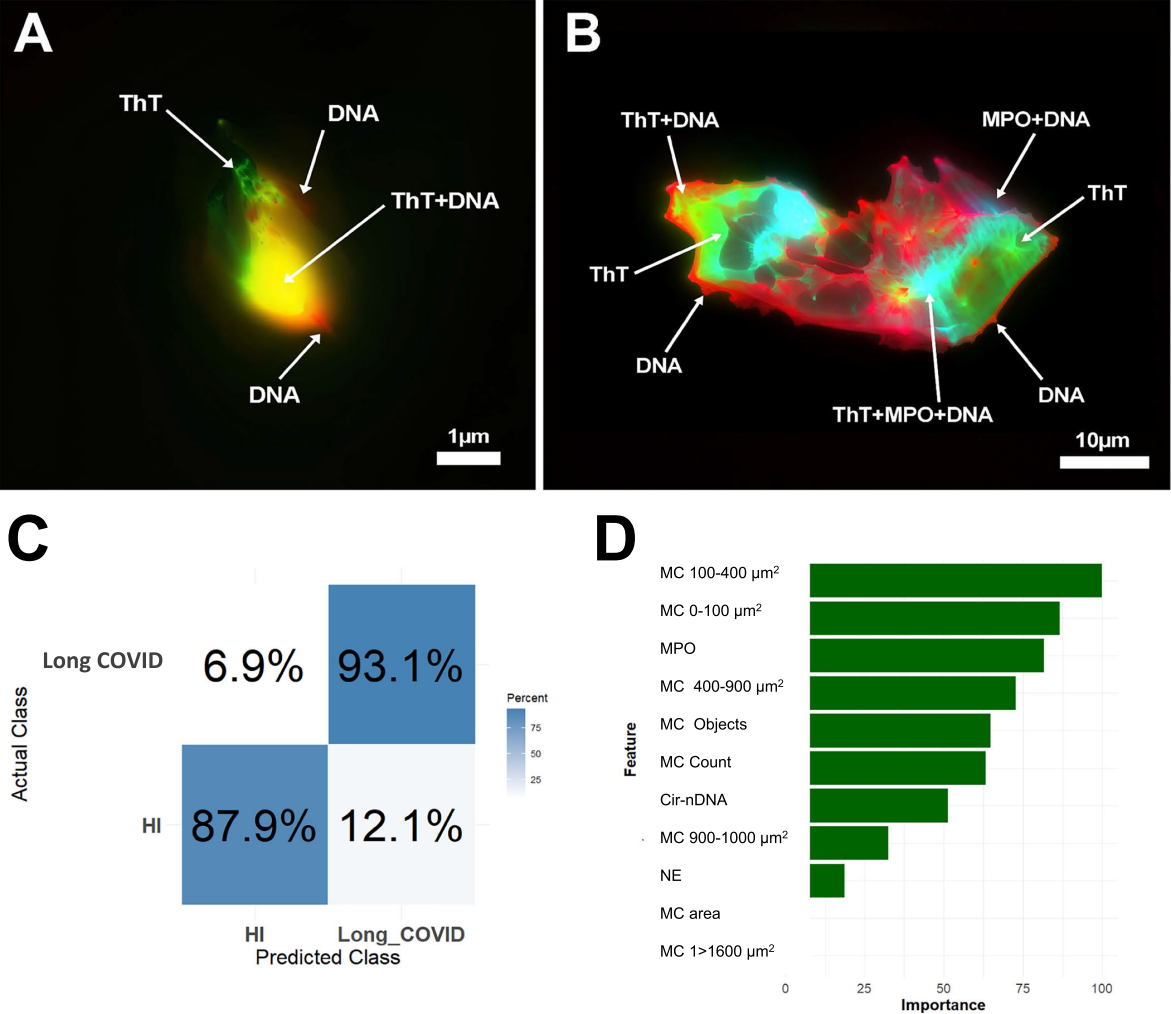
Illustrative co‐localization of NETs and microclots markers in control and LC individual plasma, and performance of LC quantitative detection performance. (A) Microclots from HI plasma and (B) a microclot apparently originating from the combination of two microclots resulting from NETs association. AI improved images from immunofluorescence microscope (Figure 3G and Fig. S7). Arrows indicate ThT localisation, DNA surrounding/covering microclot, ThT and DNA colocalization, MPO and DNA colocalization and, ThT and MPO and DNA colocalization. A and B images were subjected to a two‐step process utilizing Photoshop and Topaz Gigapixel AI; Figure 4B initial image is shown in Figure 3G and Figure S7. (C) Confusion matrix showing the performance of RF model classifying LC and healthy subjects. (D) Mean feature important scores of the cross‐validation Random Forest model for all markers were calculated. Seven markers showing high importance scores and consistent relevance across analyses were selected for the next round of model training. These markers include MPO, microclots 0–100 µm², microclots 100–400 µm², microclots 400–900 µm^2^, microclot counts, cir‐nDNA, and NE. AUC: Area under the ROC Curve; cir‐nDNA: circulating nuclear‐DNA; nuclear‐DNA; MPO, myeloperoxidase; NE, neutrophil elastase; RF: Random Forest classifier; ROC Curve: a receiver operating characteristic curve.

The characteristic NETs filaments released by activated neutrophil can be observed using fluorescence microscopy and the Hoechst DNA marker, can be seen as a diffuse light blue fluorescence, showing the chromatin outside cells (see arrows) (Figure 3A–D). We utilized staining techniques to visualize the co‐incidence of microclot formation and NETs in acute COVID‐19 and LC, compared to controls. The micrographs in Figure 3E and F highlight the use of Thioflavin T (ThT) for microclots, alongside Hoechst or SYTO stains for DNA, indicating NETs presence (Hoechst in blue, SYTO in red, and ThT in green, with a combined overlay in the third column). The bottom row (Figure 3G) shows plasma samples treated with ThT, SYTO and myeloperoxidase (MPO) stains (SYTO in red, ThT in green and MPO in blue). MPO, ThT and DNA clearly colocalized within microclots. and may also be observed alone in a few localizations in the microclots image. Arrows highlight DNA localizations, illustrating NETs surrounding or outside microclots, or between two microclots (Figure 3 bottom right image; enlargement in Figure 4B). Note, previous studies showed MPO and DNA only colocalized when neutrophils are activated [33], or within a coronary thrombus [30]. In addition, in vitro colocalization of neutrophils, extracellular DNA and coagulation factors such as fibrinogen was reported during NETosis [34].

In their structural, biological, physiological, and pathophysiological characteristics, microclots differ from traditional blood clots (or fibrin mesh formation) in several fundamental ways. Structurally, microclots tend to be smaller and are found as free entities in circulation. They also exhibit significant structural heterogeneity. Healthy fiber diameters are more uniform and clot into a gel mesh, typically only induced during a physiological clotting event such as following a cut, and under the action of thrombin and the activation of the clotting cascade. Biologically, clots formed in such ‘physiological’ circumstances are composed largely of platelets and fibrin designed to stop bleeding. By contrast microclots are characterized by an amyloid‐like nature, which can be revealed by staining them with suitable fluorogenic dyes. In addition, we have shown that microclots contain not only fibrinogen molecules, but also entrap various inflammatory molecules and even antibodies [10, 12]. An important factor initiating platelet activation and the formation of intravascular microclots is endothelial damage triggering widespread endothelial damage (endothelialitis). The presence of intravascular microclots in small blood vessels perpetuates endothelial cell damage (endothelialitis), which consequently adopt the pro‐adhesive phenotype, thus exposing those microclots to microorganisms that favour the elimination and limit the dissemination of the pathogen [35, 36].

Our fluorescence microscopy analysis (as shown in the suppl. doc) demonstrated that the addition of spike protein or LPS to purified fibrinogen induced the formation of insoluble microclots (Figure S6). Fibrin(ogen) in microclots were confirmed by binding of the Fibrinogen α‐chain antibody (Alexa Fluor 594), with amyloid regions identified by Thioflavin T (ThT) staining. This pattern was consistent across both healthy and Long COVID samples, validating the presence of fibrin(ogen) and amyloidogenic characteristics within the microclots (see Suppl Figure S6).

### 3.3 Impact of NETs and Microclots Association on LC Complications

Abnormal blood clotting and microclots formation thus appear as important factors that may explain the development and persistence of symptoms in LC. Indeed, several researchers have signalled the exacerbation by a dysregulated thromboinflammatory response of the interplay between coagulation and innate immunity [29, 30, 37, 38]. Platelet hyperactivation and endothelial dysfunction would therefore appear to be a unifying pathway underlying the wide range of symptoms seen in LC [2, 11].

Mechanistically, cell viral infection and the resulting inflammation leads to the stimulation of neutrophils, which leads in turn to the release of tissue factors and the degradation of anticoagulants by monocytes, promoting coagulation [25]. As a result, the interplay between the innate immune response and blood coagulation (immunothrombosis) leads to the formation of microthromboses [29, 37, 39].

Existing evidence points to a central role played by NETs in immunothrombosis [27, 29, 35, 39, 40]. Thus, activated platelets are an endogenous stimulus capable of inducing NETs [22, 25, 41]. NETs interact physically with platelets, both through their binding with the P‐selectin glycoprotein ligand and through the binding of the high mobility group box 1 (HMGB1) nuclear protein to the RAGE or TLR4 receptors [41]. There is also other evidence of the mechanistic link between NETs and coagulation, such as the binding of: (i) the coagulation factor fibrinogen with DNA‐rich NETs; (ii), the coagulation factors prothrombin, FX and FVIIa with activated neutrophils during NETosis; and (iii), the anticoagulant activated [42] protein C (APC) with activated neutrophils and DNA‐rich NETs [29, 43].

Neutrophils and platelets activate each other mutually, and their physical interaction constitutes a central loop of amplification of immuno‐thrombosis triggering NETs formation, (39). We speculate that the deterioration or activation of the endothelial cells by circulating microclots leads to inflammation which in turn activate neutrophils [42, 44]. Consequently, exacerbated and deleterious NETs production might be not only maintained by the NETs/thrombosis feedback loop but also by the positive NETs/inflammation feedback loop [44]. This may cause a persistent stimulation of neutrophils, which may in turn lead to the pathogenic microthrombosis phenomena observed in LC. Recent proteomic analysis showed neutrophil activation as well as neutrophil signatures in LC patients associated with clotting cascade members. Our observations point to the possibility that, in addition to this hypercoagulation state, a persistent imbalance in NETs formation, plausibly caused by viral persistence [45], may lead to a high structural association of NETs with microclots. We speculate this would impair clot lysis, contributing to microclots higher numbers in the circulation and a lower clearance level. The protective role of NETs in infection, therefore, would come at a high cost in the case of SARS‐CoV2 infection, as well as may be in certain other inflammatory diseases. However, association does not mean causation and further studies are needed to confirm our postulate.

### 3.4 Co‐Detection of Microclots and NETs Markers as a Diagnostic Test

The ability to detect these markers efficiently would pave the way for better disease monitoring, understanding the etiology of inflammation, and personalized treatment strategies. More specifically, a detection strategy that could demonstrate the combined presence of high microclot numbers and elevated levels of both NETs markers and cirDNA (either by microscopy, cytometry or ELISA) would offer an efficient means of tackling the underdiagnosis of LC, thereby improving diagnostic accuracy and appropriate intervention.

The co‐localization of MPO and DNA observed in Figure 4B aligns with their association in LC patient plasma, as shown by Spearman analysis (Figure 3D), and supports previous findings of MPO/NE/cirDNA association in COVID‐19, Long COVID, and cancer samples [17, 18, 22, 29] as well as in PMA‐stimulated neutrophil cultures where NETs formation produces NE, MPO, and cell‐free DNA [27]. These findings collectively demonstrate that concurrent elevated plasma levels of MPO, NE, and cirDNA indicate NETs formation.

To further evaluate the performance of an LC detection test which uses markers of both entities, we employed Decision Tree‐Gini, K‐nearest Neighbors Classifier (Figure S8), and Random Forests (RF) for training by machine learning. In these models, we utilized all markers for training, and five‐fold cross‐validation with 100 repetitions for each classifier, to ensure robustness, stability, and the generalizability of machine learning model performance estimates. The best performance was achieved with the RF algorithm (Figure 4C). This superior performance likely reflects the ensemble nature of RF, which reduces overfitting and improves robustness to noise and irrelevant features, making it well‐suited to moderate‐sized datasets. The mean feature importance in RF model indicates that microclots 100–400 µm², microclots 0–100 µm², MPO, microclots 400–900 µm^2^, microclot objects, microclot counts, and cirDNA all contribute to the discrimination between healthy and LC subjects (Figure 4D). RF models provide the highest performance values, with accuracy of 0.91 (95% CI: 0.90; 0.91), AUC of 0.95 (95% CI: 0.98; 0.98), sensitivity of 0.86 (95% CI: 0.86; 0.87), and specificity of 0.99 (95% CI: 0.99; 0.99). The confusion matrices show that 93% of subjects with LC and 88% of healthy individuals were correctly classified by the RF model (Figure 4C). By excluding the least important markers one by one, using a RF model we found that the most important markers for achieving the highest specificity are MPO and microclots 100–400 µm², and that these provided accuracy, AUC, sensitivity and specificity of 0.92 (95% CI: 0.91; 0.92), 0.98 (95% CI: 0.98; 0.98), 0.88 (95% CI: 0.88; 0.88) and 0.98 (95% CI: 0.98; 0.98), respectively (Figure S8). These data showed that the combination of a NETs marker (MPO) and a microclot numbering marker (100‐400 µm² size) can achieve a very high discriminating/screening power (Figure S9), as illustrated by our scatter plot presentation of MPO and 100–400 µm² microclots concentration values (Figure 2E). While promising, our preliminary data do not yet validate NETs and microclots as biomarkers for Long COVID in practical clinical settings. Additional studies with larger patient cohorts and comparisons among individuals with similar symptoms are essential for further validation.

Plasma from healthy individuals contains low numbers of microclots of low sizes as observed here (Figure 1A,B, Figure 3; and Figure S2 and S3) and previously *(7)*, but this study revealed they may be covered at least partially with NETs. We suggest that microclots are bound/entrapped by floating NET parts in the blood stream, and that physico‐chemical properties of both entities enable close structural association inhibiting their degradation in the circulation. Thus, NET parts may now be conceived as a potential microclot constituent in addition to the insoluble fibrin amyloid and other molecules like cytokines or antibodies [10, 12].

Although our study included two independent healthy control cohorts from different geographic regions, it did not include disease‐specific control groups such as individuals with diabetes, sepsis, or autoimmune disorders. As such, we cannot definitively assess the specificity of NETs and microclots elevations to Long COVID. Although co‐localization of ThT and MPO signals within microclot structures was observed by fluorescence microscopy, unfortunately an microscopy technique does not permit reliable quantification of the proportion of microclot that are ThT+ or MPO + ; such analyses would require high‐throughput single‐particle approaches like imaging flow cytometry. Such optimization and analysis will be a future research endeavour. While our findings demonstrate a strong quantitative and structural association between NETs and microclots in Long COVID patients, further comparative studies are needed to establish the discriminatory power of these biomarkers across different disease states. We explicitly recognize this as a limitation of the current study and recommend that future investigations include disease‐matched control cohorts to validate the diagnostic specificity and potential clinical utility of these markers. While our current analysis considers LC patients as a single group, this approach should be regarded only as a starting point. It is necessary to first identify measurable biological signals, such as NET‐associated microclots, which may ultimately enable patient stratification in the future. The rationale of this study is to explore whether a microclot and NETs burden can serve as a distinguishing feature which helps to define biologically distinct subtypes, potentially including a group characterized by heightened thrombo‐inflammatory or immune‐mediated endothelial injury. We therefore recognize the absence of clinical stratification as a limitation, while emphasizing that this study lays the groundwork for developing objective biomarker‐based subgrouping in future research.

### 3.5 General Conclusions

Microclots frequently appear in chronic inflammatory states and are more resistant to fibrinolysis, showing resistance even to powerful proteolytic enzymes such as trypsin [10]. We hypothesize that imbalanced NETs formation as observed in various inflammatory diseases such as COVID‐19 or LC might lead to higher microclots stability and therefore their elevated number in the circulation. Such resistance to normal breakdown mechanisms and the variability in their formation, even in the absence of typical clotting triggers such as thrombin, would indicate that microclots may have more widespread and clinically deleterious effects.

This study shows a robust association between biomarkers indicative of thromboinflammatory activity and LC. Since our study revealed that NETs may be a component of circulating microclots, we speculate that higher NETs formation promotes the stabilization of microclots in the circulation, leading to deleterious effects which (in part) may contribute to the symptoms of LC. The discovery of these biomarker linkages not only presents a possible novel diagnostic methodology but also novel therapeutic targets, offering prospects for future markedly improved clinical management. Consequently, our findings present a significant advancement in the understanding of the interactions between NETs and microclots in Long COVID.

## Author Contributions

Conceptualization: A.R.T., E.P. Methodology: C.S., B.P., T.H., A.M., E.E.P., S.T., M.W., A.T., T.U., C.V., C.P. Investigation: A.R.T., E.P., T.U., C.P. Visualization: A.R.T., E.P., C.V., T.U. Funding acquisition: A.R.T., E.P. Project administration: C.V., E.P., A.R.T. Supervision: A.R.T., E.P. Writing – original draft: A.R.T., T.H., E.P., C.V. Writing – review and editing: E.P., A.R.T., C.V., D.B.K., G.J.L.

## Conflicts of Interest

A.R.T., E.E.P., and B.P. are author of a patent: NEW METHOD TO DIAGNOSE INFLAMMATORY DISEASES 11194720 PCT application number PCT/EP2022/072147, Date of receipt 05 August 2022. A.R.T., C.V., and E.P. are an author of a patent DIAGNOSTIC METHOD FOR LONG COVID PCT application number GB2105644.5. E.P. is a founding director of Biocode Technologies, a Stellenbosch University start‐up company. S.T. and A.T. are employees of Biocode Technologies, as well as postgraduate students of E.P. All other authors: no competing interests to declare.

## Supporting information


**Figure S1:** Comparison of microclot numbering in Healthy Individuals (HI) and LC patient (LC) plasma as determined by flow cytometry across various parameters: Images represent micrographs captured from our control (n=38) and LC (n=50) cohorts using imaging flow cytometry. Images are captured using both brightfield (Ch01) and fluorescence (Ch07) imaging.
**Figure S2:** Values and median of cirDNA, MPO, NE concentration and microclots numbering in plasma of the long COVID (LC) patients and the healthy Individual (HI) cohorts. Healthy individuals (HI) from South Africa (SA) and France (EFS).
**Figure S3:** Values and median of cirDNA, MPO, NE concentration and microclots numbering in plasma of Long COVID (LC, in red) participants patients and healthy individuals (HI) from France (EFS, in green) and South Africa (SA, in blue). Overall, no or poor difference in markers values is noted between SA and EFS HI cohorts.
**Figure S4:** ROC analysis of microclots numbering vs HI (n=38).
**Figure S5:** ROC analysis pfcirDNA, MPO, and NE vs Healthy individuals (HI,n=38).
**Figure S6:** (A)Purified fibrinogen (Thermo‐Fisher, Rp43142) at 4 mg/ml exposed to 50ng/L lipopolysaccharide (final exposure concentration) followed by exposing to fibrinogenn α‐chain antibody (Alexa Fluor 594, ab216367) and Thioflavin T (ThT).( B) spike protein (final exposure concentration 100 ng/mL). (C) Platelet‐poor plasma from a Long COVID participant treated with Myeloperoxidase (MPO)(48‐1299‐42, Invitrogen, Waltham, MA, USA), fibrinogenn α‐chain antibody and Thioflavin T (ThT).
**Figure S7:** Original figure of the microclot showed in Fig. 3G and 4B. This figure was obtained as described in Material and Methods.
**Figure S8:** Performances of long COVID classification obtained KNeighbors and Decisiohn Tree.
**Figure S9:** Random Forest performances of long COVID classification depending on diffrent chosen markers.
**Figure S10:** Centrifugation Protocol Validation. Imaging flow cytometry results comparing plasma from a healthy individual prepared by 1200g for 10 minutes (HI_1200g), 3000g for 15 minutes (HI_3000g), or a two‐step process (1200g for 10 minutes followed by 16000g for 10 minutes, HI_16000g). It is established that plasma prepared following the two‐step method is cell‐free. Since HI_3000g and HI_16000g showed similar total counts and size distributions, the plasma derived from the 3000g for 15 minutes process is cell‐free. In contrast, plasma prepared at 1200g alone exhibited higher counts, indicating potential contamination with cells or debris.
**Table S1:** Characteristics of the healthy individuals from France (EFS).
**Table S2:** Demographics and co‐morbidities of our Long COVID (LC) patients and healthy individuals (HI) from both South Africa (SA) and France (EFS).
**Table S3:** Median of microclots parameters using imaging fluorocytometry in Long COVID (LC) patients and healthy individuals from both South Africa (SA) and France (EFS).
**Table S4:** Compiled data from microclot parameters, NETs markers and cirDNA in the full healthy cohort. Healthy individuals from both South Africa (SA) and France (EFS).
**Table S5:** Compiled data from microclot parameters. NETs markers and cirDNA in the healthy individual cohort from France (EFS).
**Table S6:** Compiled data from microclot parameters, NETs markers and cirDNA in the healthy individual cohort from South Africa (SA).
**Table S7:** Compiled data from HFAP (microclot) parameters, NETs markers and cirDNA in the long COVID (LC) patient cohort.
**Table S8:** P value of the differences between Long COVID (LC) patients and healthy individuals (SA + EFS), in respect to microclot numbers (Figure 2A) and NETs markers and cirDNA (Figure 2B).
**Table S9:** P values of the significance of the correlation between NETs markers, cirDNA and microclots in healthy individuals (SA + EFS). 0.00E+00corresponds to Mann‐WithneyP‐value with more than 15 decimals.
**Table S10:** P values of the significance of the correlation between NETs markers, cirDNA and microclots in patients with long COVID (LC). 0.00E+00corresponds to Mann‐Withney P‐value with more than 15 decimals.
**Table S11:** Number of individuals with one or more HFAP (microclots) in Long COVID (LC) patients and healthy individuals.

## Data Availability

The data that support the findings of this study are available on request from the corresponding author. The data are not publicly available due to privacy or ethical restrictions. All data are available in the main text or the Supporting materials. Material and data sharing agreement (signed on the 26th of July 2023, #S008426) between Stellenbosch University (SA) and INSERM (France). All data are available in the main text or the Supporting materials. Material and data sharing agreement (signed on the 26th of July 2023, #S008426) between Stellenbosch University (SA) and INSERM (France).
