# Supplementary material for: Circulating Microclots Are Structurally Associated With Neutrophil Extracellular Traps and Their Amounts Are Elevated in Long COVID Patients
Source: J Med Virol. 2025 Oct 2;97(10):e70613. doi: 10.1002/jmv.70613 (PMC12489976; doi:10.1002/jmv.70613)
Supplement: Supplementary file 1 — Figure S1: Comparison of microclot numbering in Healthy Individuals (HI) and LC patient (LC) plasma as determined by flow cytometry across various parameters: Images represent micrographs captured from our control (n=38) and LC (n=50) cohorts using imaging flow cytometry. Images are captured using both brightfield (Ch01) and fluorescence (Ch07) imaging. Figure S2: Values and median of cirDNA, MPO, NE concentration and microclots numbering in plasma of the long COVID (LC) patients and the healthy Individual (HI) cohorts. Healthy individuals (HI) from South Africa (SA) and France (EFS). Figure S3: Values and median of cirDNA, MPO, NE concentration and microclots numbering in plasma of Long COVID (LC, in red) participants patients and healthy individuals (HI) from France (EFS, in green) and South Africa (SA, in blue). Overall, no or poor difference in markers values is noted between SA and EFS HI cohorts. Figure S4: ROC analysis of microclots numbering vs HI (n=38). Figure S5: ROC analysis pfcirDNA, MPO, and NE vs Healthy individuals (HI,n=38). Figure S6: (A)Purified fibrinogen (Thermo‐Fisher, Rp43142) at 4 mg/ml exposed to 50ng/L lipopolysaccharide (final exposure concentration) followed by exposing to fibrinogenn α‐chain antibody (Alexa Fluor 594, ab216367) and Thioflavin T (ThT).( B) spike protein (final exposure concentration 100 ng/mL). (C) Platelet‐poor plasma from a Long COVID participant treated with Myeloperoxidase (MPO)(48‐1299‐42, Invitrogen, Waltham, MA, USA), fibrinogenn α‐chain antibody and Thioflavin T (ThT). Figure S7: Original figure of the microclot showed in Fig. 3G and 4B. This figure was obtained as described in Material and Methods. Figure S8: Performances of long COVID classification obtained KNeighbors and Decisiohn Tree. Figure S9: Random Forest performances of long COVID classification depending on diffrent chosen markers. Figure S10: Centrifugation Protocol Validation. Imaging flow cytometry results comparing plasma from a healthy individual [file JMV-97-e70613-s001.docx]

**ALL SUPPL. FIGURES FOR THE MANUSCRIPT**


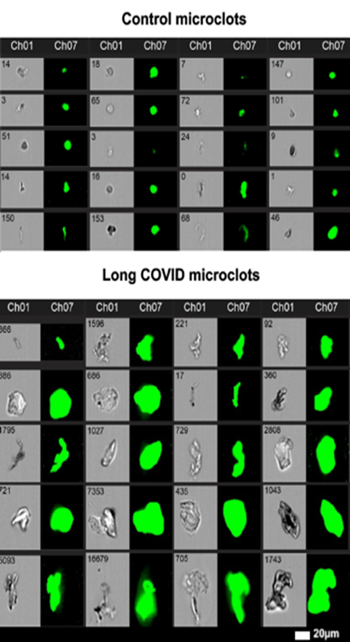


**A**

**B**

**Fig. S1. Comparison of microclot numbering in Healthy Individuals (HI) and LC patient (LC) plasma as determined by flow cytometry across various parameters: Images** represent micrographs captured from our control (n=38) and LC (n=50) cohorts using imaging flow cytometry. Images are captured using both brightfield (Ch01) and fluorescence (Ch07) imaging.

| **Median** | | | | | |
| --- | --- | --- | --- | --- | --- |
| **Cohorts** | **Cir-nDNA (ng/mL)** | **MPO (ng/mL)** | **NE (ng/mL)** | **Microclots Objects/mL** | **Microclots in 100-400µm^2^ range** |
| **(HI EFS + HI SA), n=38** | 6.3 | 10.5 | 0.8 | 3620.0 | 36.5 |
| **LC, n=50** | 36.3 | 36.6 | 11.9 | 71394.4 | 563.5 |
| **Fold from median** | 5.7 | 3.5 | 14.9 | 19.7 | 15.4 |

**Fig. S2:** Values and median of cirDNA, MPO, NE concentration and microclots numbering in plasma of the long COVID (LC) patients and the healthy Individual (HI) cohorts. Healthy individuals (HI) from South Africa (SA) and France (EFS).

| **Median** | | | | | |
| --- | --- | --- | --- | --- | --- |
| **Cohorts** | **Cir-nDNA (ng/mL)** | **MPO (ng/mL)** | **NE (ng/mL)** | **Microclots Objects/mL** | **Microclot number in 100-400µm^2^ range** |
| **HI EFS, n=24** | 7.4 | 10.5 | 1.2 | 5885.5 | 46.5 |
| **HI SA, n=14** | 5.3 | 10.5 | 0.3 | 3008.0 | 32.5 |
| **LC, n=50** | 36.3 | 3.6 | 11.9 | 71394.4 | 563.5 |
| **Fold (LC/HI EFS) from median** | 4.9 | 3.5 | 9.9 | 12.1 | 12.1 |
| **Fold (LC/HI SA) from median** | 6.8 | 3.5 | 39.7 | 23.7 | 17.3 |

**Fig. S3:** Values and median of cirDNA, MPO, NE concentration and microclots numbering in plasma of Long COVID (LC, in red) participants patients and healthy individuals (HI) from France (EFS, in green) and South Africa (SA, in blue). Overall, no or poor difference in markers values is noted between SA and EFS HI cohorts.

**A**


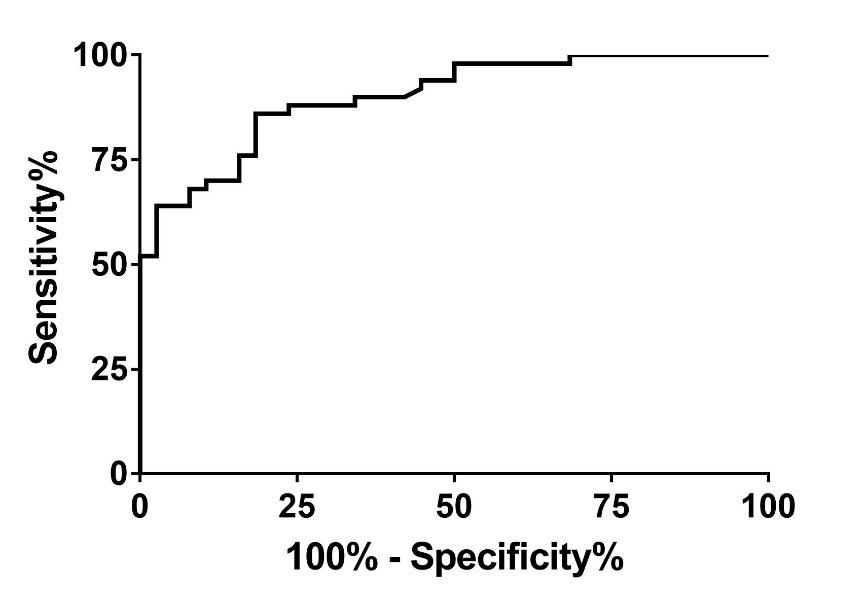


**ROC curve : cir-nDNA**

**B**


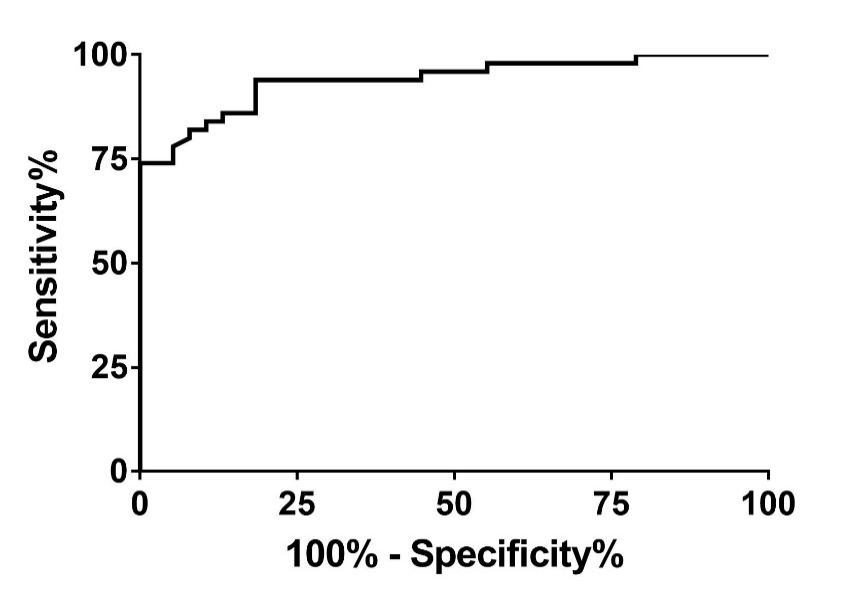


**ROC curve : MPO**

**C**


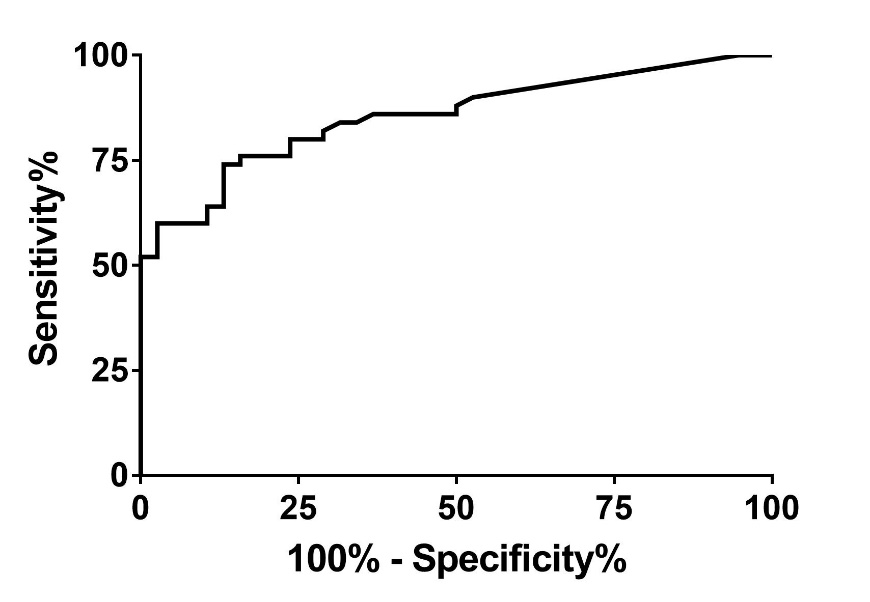


**ROC curve : NE**

**D**


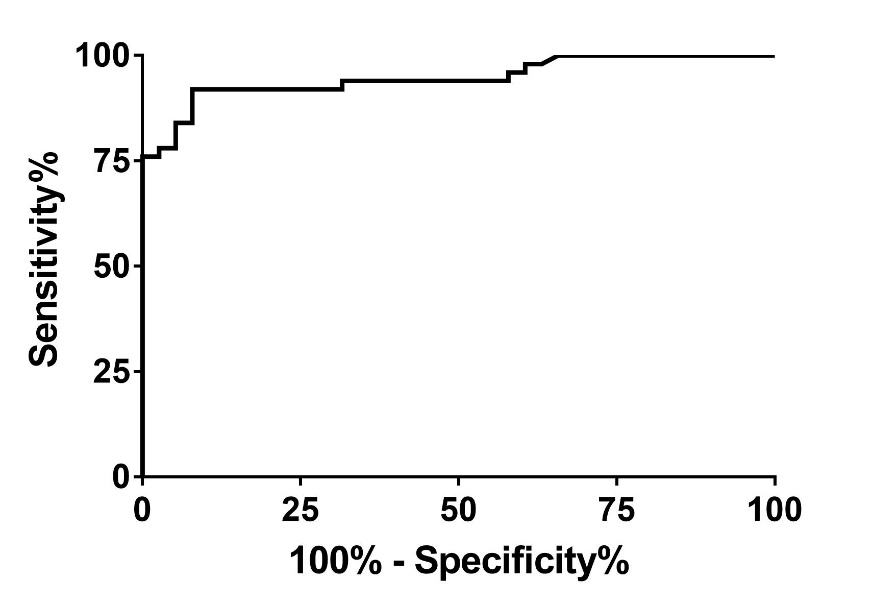


**ROC curve : cir-nDNA + MPO + NE**

AUC with 95%CI: 0.85 [0.79;0.93]

Sensitivity with 95%CI: 0.74 [0.54;0.90]

Specificity with 95%CI: 0.87 [0.74;1.00]

AUC with 95%CI: 0.95 [0.90;0.99]

Sensitivity with 95%CI: 0.92 [0.78;0.98]

Specificity with 95%CI: 0.92 [0.84;1.00]

AUC with 95%CI: 0.90 [0.84;0.96]

Sensitivity with 95%CI: 0.86 [0.62;0.96]

Specificity with 95%CI: 0.82 [0.71;1.00]

AUC with 95%CI: 0.94 [0.89;0.90]

Sensitivity with 95%CI: 0.94 [0.70;0.98]

Specificity with 95%CI: 0.82 [0.76;1.00]

**Fig. S4:** ROC analysis of microclots numbering vs HI (n=38).

**
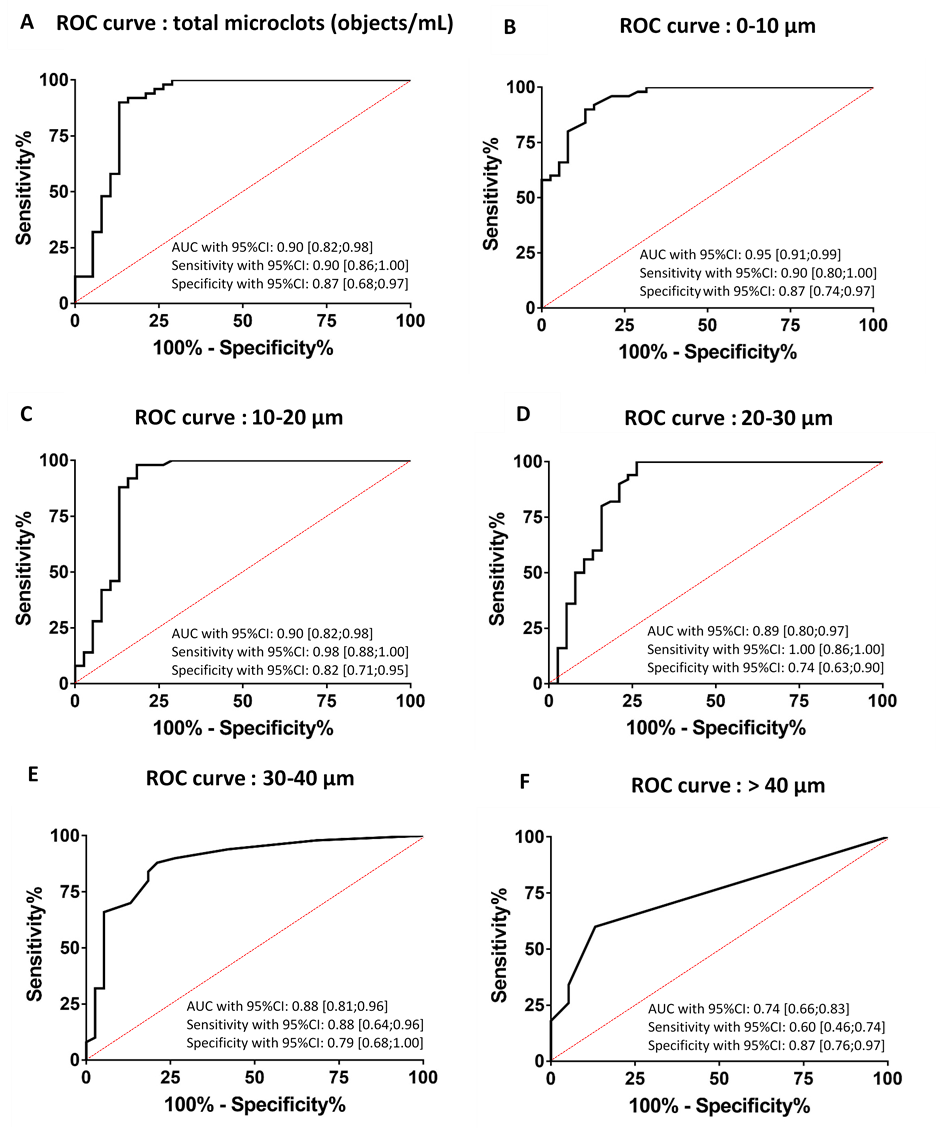
**

**
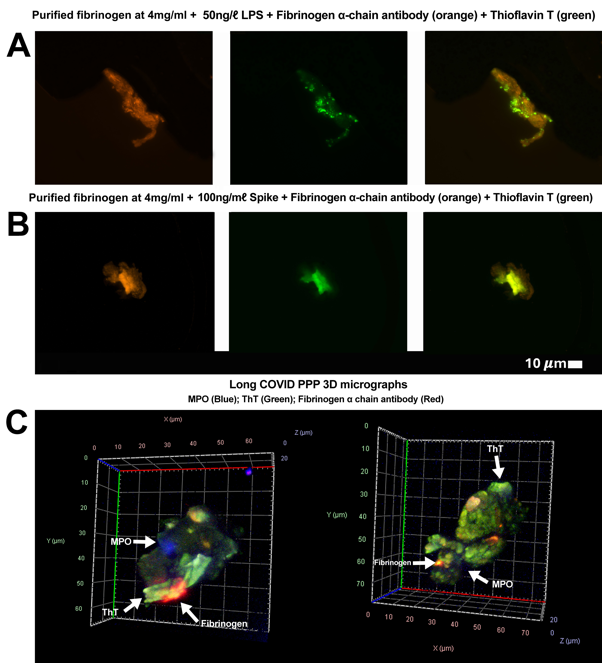
**

**Fig S6: A)** Purified fibrinogen (Thermo-Fisher, Rp43142) at 4 mg/ml exposed to 50ng/L lipopolysaccharide (final exposure concentration) followed by exposing to fibrinogenn α-chain antibody (Alexa Fluor 594, ab216367) and Thioflavin T (ThT). **B)** spike protein (final exposure concentration 100 ng/mL). C) Platelet-poor plasma from a Long COVID participant treated with Myeloperoxidase  (MPO) (48-1299-42, Invitrogen, Waltham, MA, USA), fibrinogenn α-chain antibody and Thioflavin T (ThT),

**
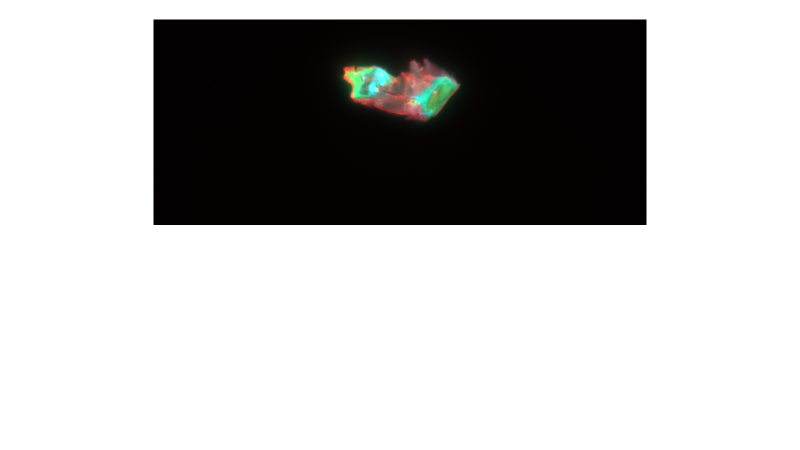
**

**Fig. S7:** Original figure of the microclot showed in Fig. 3G and 4B. This figure was obtained as described in Material and Methods

**
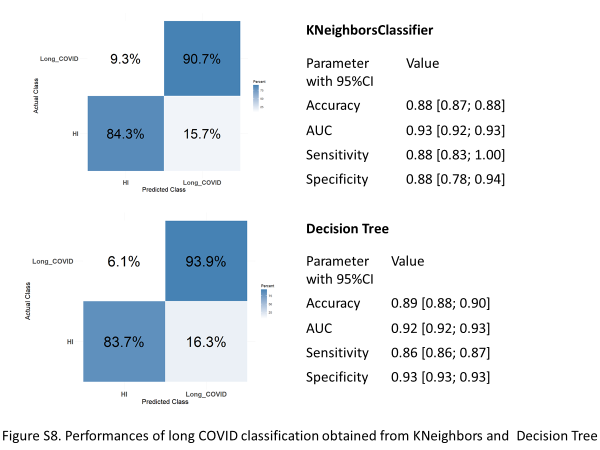
**

**
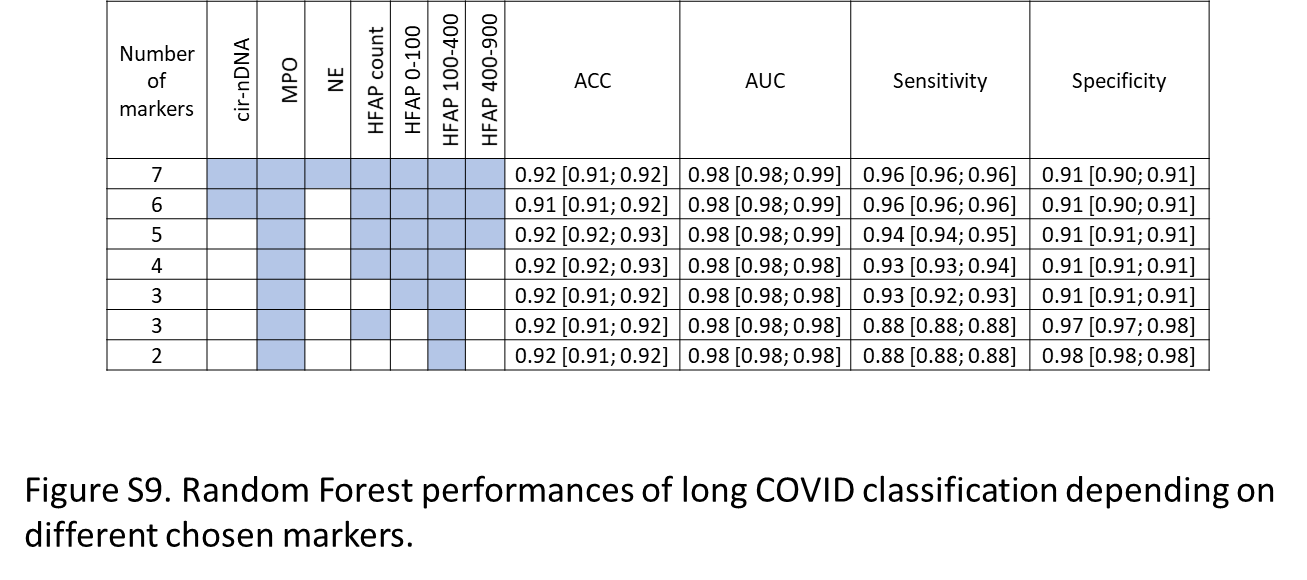
**

**A P**

**Supplementary Information S10:**

**Demonstration of the absence of cells or cell-debris in the studied samples**

1. Platelet-Poor Plasma (PPP) Preparation: PPP was prepared by centrifuging whole blood at 3000g for 15 minutes, following standard hematological protocols to create cell-free plasma (<https://brd.nci.nih.gov/brd/paper/biochem-biophys-rep/2016/pre-storage-centrifugation-conditions-have-significant-impact/127470>). This plasma was used for microscopy and imaging flow cytometry.
2. Two-Step Centrifugation for CirDNA and NETs Analysis: For the quantification of cirDNA and NETs protein markers, we used a two-step centrifugation process (1200g for 10 minutes, then 16000g for 10 minutes), which has been shown to produce plasma free of cells and debris, as indicated in published guidelines (PMID: 24658074, PMID: 30792266, PMID: 28400427, PMID: 39303668, PMID: 35198886, PMID: 38660409, PMID: 36443816, PMID: 36226380, PMID: 35198886).
3. Consistency Across Cohorts: To avoid bias, we analyzed cohorts from both France and South Africa (see Materials and Methods). Equivalent observations were made in microscopy, imaging flow cytometry, and NETs markers across both cohorts, regardless of whether plasma was prepared using the 3000g or two-step centrifugation process, confirming the absence of cell contamination.
4. Centrifugation Protocol Validation: We conducted additional experiments comparing plasma prepared by 1200g for 10 minutes with plasma prepared by our standard methods (3000g for 15 minutes and the two-step process) using an advanced imaging flow cytometer. Results showed that:
   - Plasma prepared by 3000g and the two-step process yielded similar total counts and size distributions, indicating that they are cell-free.
   - Plasma prepared at 1200g showed higher counts, suggesting the presence of cells or debris.

See below Fig.S10


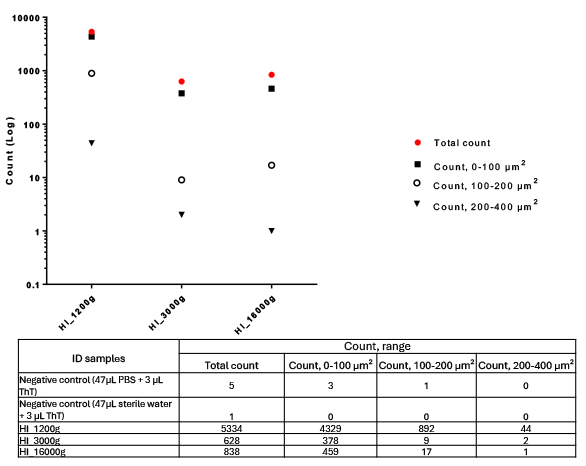


**Fig. S10:** Centrifugation Protocol Validation. Imaging flow cytometry results comparing plasma from a healthy individual prepared by 1200g for 10 minutes (HI_1200g), 3000g for 15 minutes (HI_3000g), or a two-step process (1200g for 10 minutes followed by 16000g for 10 minutes, HI_16000g).

It is established that plasma prepared following the two-step method is cell -free. Since HI_3000g and HI_16000g showed similar total counts and size distributions, the plasma derived from the 3000g for 15 minutes process is cell-free. In contrast, plasma prepared at 1200g alone exhibited higher counts, indicating potential contamination with cells or debris.

**ALL SUPPL. TABLES FOR THE MANUSCRIPT**

| **ID Sample** | **Age** | **Sex** | **Group** | **Rhesus (D)** | **Hematocrit level** |
| --- | --- | --- | --- | --- | --- |
| **EFS 1** | 59 | M | O | + | 50 |
| **EFS 2** | 25 | M | O | + | 43 |
| **EFS 3** | 64 | M | AB | + | 48 |
| **EFS 4** | 60 | M | A | + | 49 |
| **EFS 5** | 63 | F | AB | + | 40 |
| **EFS 6** | 35 | F | O | + | 43 |
| **EFS 7** | 35 | M | A | + | 47 |
| **EFS 8** | 59 | M | A | - | 43 |
| **EFS 9** | 56 | M | A | - | 47 |
| **EFS 10** | 37 | F | O | + | 44 |
| **EFS 11** | 58 | F | O | + | 38 |
| **EFS 12** | 41 | F | A | + | 43 |
| **EFS 13** | 61 | M | O | + | 47 |
| **EFS 14** | 35 | M | AB | + | 42 |
| **EFS 15** | 37 | M | B | + | 42 |
| **EFS 16** | 60 | F | B | - | 47 |
| **EFS 17** | 19 | F | O | + | 43 |
| **EFS 18** | 49 | M | O | + | 48 |
| **EFS 19** | 35 | F | O | + | 44 |
| **EFS 20** | 42 | M | O | + | 47 |
| **EFS 21** | 32 | F | B | - | 44 |
| **EFS 22** | 53 | M | A | - | 47 |
| **EFS 23** | 63 | M | O | + | 48 |
| **EFS 24** | 25 | M | O | + | 44 |

|  | **Cohort of Healthy Individuals (EFS, n=24)** | | **Age** | |
| --- | --- | --- | --- | --- |
|  | **Number** | **(%)** | **Range** | **Median** |
| **Female (F)** | 9 | 37.5% | (19-63) | 37 |
| **Male (M)** | 15 | 62.5% | (25-64) | 53 |

**Table S1:** Characteristics of the healthy individuals from France (EFS).

| **Age (represented by median [Q1-Q3])** | | | |
| --- | --- | --- | --- |
| Median age of SA healthy individuals (HI, SA) **(n = 14)** | | 28 (26-42) | |
| Median age of French healthy individuals (HI, EFS) **(n = 24)** | | 45.5 (35-59.8) | |
| Median age of Long COVID patients **(n = 50)** | | 50 (39-62) | |
| **Sex** | | | |
| Sex of SA healthy individuals (HI, SA) **(n = 14)** | | 7 males; 7 females | |
| Sex of French healthy individuals (HI, EFS) **(n = 24)** | | 15 males; 9 females | |
| Sex of Long COVID patients (LC) **(n = 50)** | | 18 males; 32 females | |
| **Vaccination status of participants before blood collection** | | | |
| SA healthy individuals (HI, SA) **(n = 14)** | | 14 Vaccinated | |
| French healthy individuals (HI, EFS) **(n = 24)** | | 24 Vaccinated | |
| Long COVID patients (LC) **(n = 50)** | | 45 Vaccinated; 4 Unvaccinated; 1 Unknown | |
| **Persistent symptoms in Long COVID (LC) patients (n = 50)** | | | |
| **Symptom** | | **% in 50 Long COVID (LC) patients with symptom** | |
| Brain fog/Concentration issues | | 78% | |
| Constant fatigue | | 74% | |
| Depression/Anxiety | | 54% | |
| Joint and muscle pain | | 50% | |
| Sleep apnea | | 48% | |
| Shortness of breath | | 42% | |
| Heart palpitations | | 38% | |
| Digestive problems | | 32% | |
| Migraines | | 24% | |
| Recurring chest pain | | 22% | |
| Vertigo/Dizziness | | 20% | |
| Low oxygen levels | | 18% | |
| Post-exertional malaise/fatigue | | 10% | |
| Parasthesia/numbness | | 10% | |
| Tinnitis | | 6% | |
| Ageusia (loss of taste) | | 4% | |
| Anosmia (loss of smell) | | 4% | |
| Kidney complications | | 2% | |
| **Co-morbidities of Long COVID (LC) patients (n = 50) vs healthy individuals from SA (HI SA, n = 14) vs healthy individuals from EFS (HI EFS, n = 24)** | | | |
| **Co-morbidity** | **% in 50 Long COVID (LV) Patients** | **% in 14 SA healthy individuals (HI, SA)** | **% in 24 EFS healthy individuals (HI, EFS)** |
| Hyperlipidaemia | 24% | 0% | 0% |
| Hypertension | 22% | 0% | 0% |
| Gut dysbiosis | 10% | 0% | 0% |
| Peridontitis or gingivitis | 8% | 0% | 0% |
| Rheumatoid Arthritis | 8% | 0% | 0% |
| Cancer | 6% | 0% | 0% |
| Rosacea | 6% | 0% | 0% |
| Type 2 Diabetes | 6% | 0% | 0% |
| Type 1 Diabetes | 4% | 0% | 0% |
| Cardiovascular disease | 4% | 0% | 0% |
| Chronic obstructive pulmonary disease | 2% | 0% | 0% |

**Table S*2***: Demographics and co-morbidities of our Long COVID (LC) patients and healthy individuals (HI) from both South Africa (SA) and France (EFS).

| **Median of microclot parameters as determined by imaging cytofluorometry** | | | | | | | | |
| --- | --- | --- | --- | --- | --- | --- | --- | --- |
| **Cohorts** | **total count** | **Objects/mL** | **Mean Area (µm²)** | **Count**  **in 0-100µm^2^**  **range** | **Count**  **in 100-400µm^2^**  **range** | **Count**  **in 400-900µm^2^**  **range** | **Count**  **in 900-1600µm^2^**  **range** | **Count**  **in > 1600µm^2^**  **range** |
| **HI EFS, n=24** | 76.5 | 5885.5 | 289.4 | 3.0 | 46.5 | 11.5 | 1.5 | 0.0 |
| **HI SA, n=14** | 39.0 | 3008.0 | 207.2 | 5.0 | 32.5 | 2.5 | 0.5 | 0.0 |
| **(HI EFS + HI SA), n=38** | 47.0 | 3620.0 | 258.5 | 4.0 | 36.5 | 5.0 | 1.0 | 0.0 |
| **LC, n=50** | 928.5 | 71394.4 | 261.4 | 97.5 | 563.5 | 122.0 | 14.0 | 1.0 |

**Table S3:** Median of microclots parameters using imaging fluorocytometry in Long COVID (LC) patients and healthy individuals from both South Africa (SA) and France (EFS).

| **ID Sample** | **cirDNA (ng/mL)** | **MPO (ng/mL)** | **NE (ng/mL)** | **Microclot**  **total count** | **Microclot**  **Objects/mL** | **Microclot**  **Mean Area (µm²)** | **Microclot**  **count in 0-100µm² range** | **Microclot**  **count in 100-400µm^2^ range** | **Microclot**  **count in 400-900µm^2^ range** | **Microclot**  **count in 900-1600µm^2^ range** | **Microclot**  **count in > 1600µm^2^ range** |
| --- | --- | --- | --- | --- | --- | --- | --- | --- | --- | --- | --- |
| **EFS 1** | 10.6 | 11.8 | 1.6 | 45 | 3466 | 285 | 3 | 34 | 6 | 1 | 1 |
| **EFS 2** | 14.8 | 11.0 | 0.6 | 25 | 1925 | 318 | 2 | 17 | 5 | 0 | 1 |
| **EFS 3** | 18.3 | 12.7 | 1.1 | 18 | 1382 | 263 | 3 | 11 | 3 | 1 | 0 |
| **EFS 4** | 7.5 | 20.7 | 2.9 | 47 | 3619 | 207 | 8 | 35 | 3 | 1 | 0 |
| **EFS 5** | 13.1 | 15.5 | 3.1 | 4151 | 320645 | 233 | 46 | 3877 | 226 | 2 | 0 |
| **EFS 6** | 2.3 | 6.1 | 1.3 | 736 | 56679 | 283 | 1 | 642 | 90 | 3 | 0 |
| **EFS 7** | 2.3 | 15.6 | 4.7 | 170 | 13092 | 445 | 4 | 84 | 75 | 7 | 0 |
| **EFS 8** | 2.4 | 14.2 | 6.8 | 192 | 14831 | 294 | 11 | 145 | 30 | 6 | 0 |
| **EFS 9** | 2.4 | 10.8 | 0.3 | 108 | 8317 | 439 | 2 | 55 | 44 | 7 | 0 |
| **EFS 10** | 9.6 | 5.5 | 0.3 | 165 | 12707 | 229 | 30 | 114 | 19 | 2 | 0 |
| **EFS 11** | 13.5 | 7.9 | 0.3 | 13 | 1004 | 366 | 1 | 7 | 5 | 0 | 0 |
| **EFS 12** | 2.1 | 11.8 | 0.3 | 27 | 2072 | 299 | 5 | 16 | 6 | 0 | 0 |
| **EFS 13** | 3.0 | 10.0 | 1.1 | 2056 | 158331 | 324 | 5 | 1619 | 404 | 25 | 3 |
| **EFS 14** | 7.2 | 10.3 | 4.0 | 948 | 72982 | 267 | 3 | 823 | 115 | 7 | 0 |
| **EFS 15** | 8.3 | 6.7 | 1.3 | 3864 | 297472 | 408 | 2 | 2277 | 1502 | 80 | 3 |
| **EFS 16** | 3.6 | 17.9 | 2.1 | 100 | 7703 | 230 | 10 | 79 | 11 | 0 | 0 |
| **EFS 17** | 35.5 | 14.1 | 8.8 | 10 | 770 | 358 | 4 | 2 | 3 | 1 | 0 |
| **EFS 18** | 1.8 | 9.5 | 0.2 | 21 | 1617 | 239 | 3 | 16 | 1 | 1 | 0 |
| **EFS 19** | 25.8 | 8.4 | 0.3 | 127 | 9747 | 414 | 0 | 78 | 43 | 6 | 0 |
| **EFS 20** | 22.6 | 9.5 | 3.9 | 41 | 3167 | 318 | 1 | 27 | 12 | 1 | 0 |
| **EFS 21** | 20.5 | 13.0 | 3.9 | 101 | 7778 | 260 | 19 | 66 | 12 | 4 | 0 |
| **EFS 22** | 1.5 | 8.3 | 0.3 | 30 | 2317 | 257 | 3 | 21 | 5 | 1 | 0 |
| **EFS 23** | 1.5 | 9.5 | 0.3 | 34 | 2626 | 194 | 7 | 24 | 3 | 0 | 0 |
| **EFS 24** | 3.2 | 9.5 | 0.3 | 53 | 4068 | 384 | 3 | 38 | 9 | 2 | 1 |
| **SA 1** | 1.0 | 7.9 | 0.3 | 150 | 11551 | 153 | 64 | 80 | 5 | 1 | 0 |
| **SA 2** | 4.6 | 6.7 | 0.3 | 23 | 1772 | 337 | 4 | 12 | 5 | 2 | 0 |
| **SA 3** | 25.5 | 20.0 | 11.2 | 47 | 3621 | 200 | 5 | 40 | 2 | 0 | 0 |
| **SA 4** | 2.7 | 11.2 | 0.3 | 29 | 2115 | 270 | 5 | 20 | 2 | 2 | 0 |
| **SA 5** | 20.6 | 18.4 | 9.2 | 43 | 3312 | 186 | 4 | 37 | 2 | 0 | 0 |
| **SA 6** | 13.5 | 23.1 | 1.0 | 78 | 5987 | 139 | 17 | 61 | 0 | 0 | 0 |
| **SA 7** | 15.1 | 13.3 | 0.2 | 73 | 5603 | 206 | 11 | 53 | 6 | 3 | 0 |
| **SA 8** | 9.4 | 9.8 | 0.3 | 69 | 5314 | 203 | 9 | 54 | 4 | 2 | 0 |
| **SA 9** | 1.4 | 20.5 | 9.3 | 14 | 1078 | 245 | 1 | 11 | 2 | 0 | 0 |
| **SA 10** | 13.8 | 6.3 | 0.3 | 55 | 4236 | 185 | 13 | 36 | 6 | 0 | 0 |
| **SA 11** | 4.4 | 7.5 | 0.3 | 19 | 1458 | 215 | 1 | 16 | 2 | 0 | 0 |
| **SA 12** | 5.4 | 6.5 | 0.3 | 35 | 2704 | 215 | 2 | 29 | 3 | 1 | 0 |
| **SA 13** | 1.2 | 5.4 | 0.3 | 35 | 2686 | 208 | 5 | 27 | 2 | 1 | 0 |
| **SA 14** | 5.3 | 23.2 | 2.3 | 20 | 1541 | 235 | 1 | 16 | 3 | 0 | 0 |
| **HI EFS and HI SA, median (n=38)** | **6.3** | **10.5** | **0.8** | **47.0** | **3620.0** | **258.5** | **4.0** | **36.5** | **5.0** | **1.0** | **0.0** |

**Table S4:** Compiled data from microclot parameters, NETs markers and cirDNA in the full healthy cohort. Healthy individuals from both South Africa (SA) and France (EFS)

| **ID Sample** | **cirDNA (ng/mL)** | **MPO (ng/mL)** | **NE (ng/mL)** | **Microclot**  **total count** | **Microclot**  **Objects/mL** | **Microclot**  **Mean Area (µm²)** | **Microclot**  **count in 0-100µm^2^ range** | **Microclot**  **count in 100-400µm^2^ range** | **Microclot**  **count in 400-900µm^2^ range** | **Microclot**  **count**  **In 900-1600µm^2^ range** | **Microclot**  **count**  **in > 1600µm^2^**  **range** |
| --- | --- | --- | --- | --- | --- | --- | --- | --- | --- | --- | --- |
| **EFS 1** | 10.6 | 11.8 | 1.6 | 45 | 3466 | 285 | 3 | 34 | 6 | 1 | 1 |
| **EFS 2** | 14.8 | 11.0 | 0.6 | 25 | 1925 | 318 | 2 | 17 | 5 | 0 | 1 |
| **EFS 3** | 18.3 | 12.7 | 1.1 | 18 | 1382 | 263 | 3 | 11 | 3 | 1 | 0 |
| **EFS 4** | 7.5 | 20.7 | 2.9 | 47 | 3619 | 207 | 8 | 35 | 3 | 1 | 0 |
| **EFS 5** | 13.1 | 15.5 | 3.1 | 4151 | 320645 | 233 | 46 | 3877 | 226 | 2 | 0 |
| **EFS 6** | 2.3 | 6.1 | 1.3 | 736 | 56679 | 283 | 1 | 642 | 90 | 3 | 0 |
| **EFS 7** | 2.3 | 15.6 | 4.7 | 170 | 13092 | 445 | 4 | 84 | 75 | 7 | 0 |
| **EFS 8** | 2.4 | 14.2 | 6.8 | 192 | 14831 | 294 | 11 | 145 | 30 | 6 | 0 |
| **EFS 9** | 2.4 | 10.8 | 0.3 | 108 | 8317 | 439 | 2 | 55 | 44 | 7 | 0 |
| **EFS 10** | 9.6 | 5.5 | 0.3 | 165 | 12707 | 229 | 30 | 114 | 19 | 2 | 0 |
| **EFS 11** | 13.5 | 7.9 | 0.3 | 13 | 1004 | 366 | 1 | 7 | 5 | 0 | 0 |
| **EFS 12** | 2.1 | 11.8 | 0.3 | 27 | 2072 | 299 | 5 | 16 | 6 | 0 | 0 |
| **EFS 13** | 3.0 | 10.0 | 1.1 | 2056 | 158331 | 324 | 5 | 1619 | 404 | 25 | 3 |
| **EFS 14** | 7.2 | 10.3 | 4.0 | 948 | 72982 | 267 | 3 | 823 | 115 | 7 | 0 |
| **EFS 15** | 8.3 | 6.7 | 1.3 | 3864 | 297472 | 408 | 2 | 2277 | 1502 | 80 | 3 |
| **EFS 16** | 3.6 | 17.9 | 2.1 | 100 | 7703 | 230 | 10 | 79 | 11 | 0 | 0 |
| **EFS 17** | 35.5 | 14.1 | 8.8 | 10 | 770 | 358 | 4 | 2 | 3 | 1 | 0 |
| **EFS 18** | 1.8 | 9.5 | 0.2 | 21 | 1617 | 239 | 3 | 16 | 1 | 1 | 0 |
| **EFS 19** | 25.8 | 8.4 | 0.3 | 127 | 9747 | 414 | 0 | 78 | 43 | 6 | 0 |
| **EFS 20** | 22.6 | 9.5 | 3.9 | 41 | 3167 | 318 | 1 | 27 | 12 | 1 | 0 |
| **EFS 21** | 20.5 | 13.0 | 3.9 | 101 | 7778 | 260 | 19 | 66 | 12 | 4 | 0 |
| **EFS 22** | 1.5 | 8.3 | 0.3 | 30 | 2317 | 257 | 3 | 21 | 5 | 1 | 0 |
| **EFS 23** | 1.5 | 9.5 | 0.3 | 34 | 2626 | 194 | 7 | 24 | 3 | 0 | 0 |
| **EFS 24** | 3.2 | 9.5 | 0.3 | 53 | 4068 | 384 | 3 | 38 | 9 | 2 | 1 |
| **EFS HI median (n=24)** | 7.4 | 10.5 | 1.2 | 76.5 | 5885.5 | 289.4 | 3.0 | 46.5 | 11.5 | 1.5 | 0.0 |

**Table S5:** Compiled data from microclot parameters. NETs markers and cirDNA in the healthy individual cohort from France (EFS).

| **ID sample** | **cirDNA (ng/mL)** | **MPO (ng/mL)** | **NE (ng/mL)** | **Microclot**  **total count** | **Microclot**  **Objects/mL** | **Microclot**  **Mean Area**  **(µm²)** | **Microclot**  **count**  **in 0-100µm^2^**  **range** | **Microclot**  **count**  **in 100-400µm^2^**  **range** | **Microclot**  **count**  **in 400-900µm^2^**  **range** | **Microclot**  **count**  **in 900-1600µm^2^**  **range** | **Microclot**  **count**  **in > 1600µm^2^**  **range** |
| --- | --- | --- | --- | --- | --- | --- | --- | --- | --- | --- | --- |
| **SA 1** | 1.0 | 7.9 | 0.3 | 150 | 11551 | 153 | 64 | 80 | 5 | 1 | 0 |
| **SA 2** | 4.6 | 6.7 | 0.3 | 23 | 1772 | 337 | 4 | 12 | 5 | 2 | 0 |
| **SA 3** | 25.5 | 20.0 | 11.2 | 47 | 3621 | 200 | 5 | 40 | 2 | 0 | 0 |
| **SA 4** | 2.7 | 11.2 | 0.3 | 29 | 2115 | 270 | 5 | 20 | 2 | 2 | 0 |
| **SA 5** | 20.6 | 18.4 | 9.2 | 43 | 3312 | 186 | 4 | 37 | 2 | 0 | 0 |
| **SA 6** | 13.5 | 23.1 | 1.0 | 78 | 5987 | 139 | 17 | 61 | 0 | 0 | 0 |
| **SA 7** | 15.1 | 13.3 | 0.2 | 73 | 5603 | 206 | 11 | 53 | 6 | 3 | 0 |
| **SA 8** | 9.4 | 9.8 | 0.3 | 69 | 5314 | 203 | 9 | 54 | 4 | 2 | 0 |
| **SA 9** | 1.4 | 20.5 | 9.3 | 14 | 1078 | 245 | 1 | 11 | 2 | 0 | 0 |
| **SA 10** | 13.8 | 6.3 | 0.3 | 55 | 4236 | 185 | 13 | 36 | 6 | 0 | 0 |
| **SA 11** | 4.4 | 7.5 | 0.3 | 19 | 1458 | 215 | 1 | 16 | 2 | 0 | 0 |
| **SA 12** | 5.4 | 6.5 | 0.3 | 35 | 2704 | 215 | 2 | 29 | 3 | 1 | 0 |
| **SA 13** | 1.2 | 5.4 | 0.3 | 35 | 2686 | 208 | 5 | 27 | 2 | 1 | 0 |
| **SA 14** | 5.3 | 23.2 | 2.3 | 20 | 1541 | 235 | 1 | 16 | 3 | 0 | 0 |
| **SA HI**   **median (n=14)** | **5.3** | **10.5** | **0.3** | **39.0** | **3008.0** | **207.2** | **5.0** | **32.5** | **2.5** | **0.5** | **0.0** |

**Table S6:** Compiled data from microclot parameters, NETs markers and cirDNA in the healthy individual cohort from South Africa (SA).

| **ID Sample** | **CirDNA (ng/ml)** | **MPO (ng/ml)** | **NE (ng/ml)** | **Microclot**  **total count** | **Microclot**  **Objects/mL** | **Microclot**  **Mean Area (µm²)** | **Microclot**  **count in 0-100µm^2^ range** | **Microclot**  **count in 100-400µm^2^ range** | **Microclot**  **count in 400-900µm^2^ range** | **Microclot**  **count in 900-1600µm^2^ range** | **Microclot**  **count in > 1600µm^2^ range** |
| --- | --- | --- | --- | --- | --- | --- | --- | --- | --- | --- | --- |
| **LC1** | 2.8 | 11.1 | 0.3 | 393 | 29962 | 289 | 16 | 295 | 71 | 11 | 0 |
| **LC2** | 20 | 38.8 | 16.4 | 8228 | 631507 | 271 | 258 | 6705 | 1151 | 113 | 1 |
| **LC3** | 37.8 | 46.4 | 19.9 | 1518 | 114966 | 266 | 97 | 1182 | 224 | 14 | 1 |
| **LC4** | 36.1 | 36.9 | 13.4 | 1443 | 110014 | 83 | 1085 | 317 | 39 | 2 | 0 |
| **LC5** | 40.2 | 22.6 | 6.3 | 812 | 62322 | 202 | 260 | 462 | 80 | 10 | 0 |
| **LC6** | 45.6 | 39.9 | 6.9 | 931 | 71717 | 336 | 27 | 656 | 218 | 27 | 3 |
| **LC7** | 16.9 | 46.7 | 27.6 | 2003 | 153218 | 314 | 45 | 1552 | 344 | 58 | 4 |
| **LC8** | 217.7 | 33.3 | 9.4 | 2231 | 171231 | 324 | 114 | 1622 | 407 | 79 | 9 |
| **LC9** | 22.4 | 40.7 | 3.3 | 2203 | 169082 | 321 | 113 | 1544 | 470 | 73 | 3 |
| **LC10** | 41.2 | 42.5 | 11.9 | 2400 | 184878 | 146 | 1203 | 1044 | 140 | 12 | 1 |
| **LC11** | 22.8 | 25.8 | 5.9 | 268 | 20364 | 247 | 19 | 212 | 34 | 2 | 1 |
| **LC12** | 62.1 | 87.1 | 41.3 | 375 | 28694 | 261 | 20 | 299 | 46 | 10 | 0 |
| **LC13** | 156.7 | 80.8 | 114.4 | 5548 | 425814 | 158 | 2369 | 2832 | 326 | 21 | 0 |
| **LC14** | 95 | 65.7 | 88.8 | 8297 | 620169 | 212 | 1931 | 5452 | 855 | 59 | 0 |
| **LC15** | 36.5 | 45 | 14.1 | 741 | 56120 | 204 | 219 | 441 | 74 | 7 | 0 |
| **LC16** | 179.4 | 32.1 | 20.2 | 3218 | 247891 | 279 | 164 | 2512 | 469 | 69 | 4 |
| **LC17** | 32.9 | 35.5 | 0.6 | 2150 | 165620 | 245 | 208 | 1646 | 276 | 20 | 0 |
| **LC18** | 56.7 | 49.3 | 35.5 | 2451 | 188807 | 243 | 325 | 1779 | 317 | 29 | 1 |
| **LC19** | 38.4 | 41.7 | 29.0 | 824 | 63051 | 143 | 452 | 321 | 46 | 5 | 0 |
| **LC20** | 85 | 41.4 | 12.0 | 1222 | 93790 | 247 | 154 | 880 | 169 | 17 | 2 |
| **LC21** | 12.4 | 77.1 | 34.6 | 247 | 18837 | 318 | 20 | 163 | 56 | 7 | 1 |
| **LC22** | 6.1 | 62.6 | 22.1 | 310 | 23873 | 235 | 33 | 242 | 30 | 5 | 0 |
| **LC23** | 6.6 | 9.9 | 0.3 | 147 | 11320 | 242 | 11 | 119 | 16 | 1 | 0 |
| **LC24** | 18 | 17.7 | 3.4 | 656 | 50349 | 275 | 55 | 489 | 100 | 11 | 1 |
| **LC25** | 8.3 | 20.1 | 0.8 | 248 | 19034 | 273 | 87 | 107 | 40 | 12 | 2 |
| **LC26** | 51.5 | 46.7 | 34.4 | 3199 | 244706 | 287 | 387 | 2167 | 572 | 69 | 4 |
| **LC27** | 194.2 | 33.7 | 57.7 | 5445 | 417909 | 333 | 72 | 3988 | 1297 | 81 | 7 |
| **LC28** | 19 | 70.1 | 24.0 | 537 | 40805 | 351 | 20 | 381 | 104 | 27 | 5 |
| **LC29** | 16.6 | 21.2 | 5.8 | 161 | 12357 | 197 | 17 | 131 | 13 | 0 | 0 |
| **LC30** | 34.8 | 26.2 | 17.9 | 836 | 63106 | 293 | 98 | 552 | 161 | 24 | 1 |
| **LC31** | 14 | 26.6 | 5.7 | 1268 | 96351 | 379 | 32 | 863 | 272 | 90 | 11 |
| **LC32** | 8.1 | 7.7 | 0.3 | 179 | 13561 | 315 | 29 | 108 | 35 | 6 | 1 |
| **LC33** | 19.3 | 17.8 | 23.5 | 454 | 34508 | 313 | 11 | 337 | 92 | 14 | 0 |
| **LC34** | 43.7 | 53.2 | 23.4 | 1584 | 121982 | 260 | 119 | 1263 | 181 | 18 | 3 |
| **LC35** | 114.2 | 25.1 | 0.3 | 108 | 8289 | 282 | 8 | 78 | 20 | 1 | 1 |
| **LC36** | 25.9 | 25.1 | 6.0 | 118 | 9087 | 254 | 6 | 94 | 15 | 3 | 0 |
| **LC37** | 17.5 | 17.5 | 10.0 | 651 | 49813 | 245 | 86 | 470 | 89 | 6 | 0 |
| **LC38** | 16.5 | 46.1 | 10.1 | 3804 | 290897 | 173 | 1790 | 1638 | 327 | 47 | 2 |
| **LC39** | 23.6 | 24.1 | 2.6 | 4692 | 360115 | 152 | 2294 | 2066 | 307 | 24 | 1 |
| **LC40** | 38.5 | 20.7 | 4.1 | 326 | 25097 | 262 | 23 | 253 | 44 | 6 | 0 |
| **LC41** | 35.1 | 32.9 | 33.4 | 2464 | 189114 | 208 | 546 | 1668 | 231 | 19 | 0 |
| **LC42** | 26.2 | 20.6 | 1.6 | 1210 | 92586 | 426 | 21 | 737 | 357 | 80 | 15 |
| **LC43** | 527.5 | 109.8 | 83.3 | 4947 | 369989 | 290 | 170 | 3908 | 742 | 120 | 7 |
| **LV44** | 109 | 16.4 | 0.3 | 679 | 52114 | 200 | 136 | 486 | 51 | 6 | 0 |
| **LV45** | 100.5 | 46.3 | 11.1 | 1219 | 93559 | 154 | 566 | 575 | 74 | 4 | 0 |
| **LC46** | 63.7 | 80.9 | 30.2 | 3810 | 292421 | 179 | 1226 | 2280 | 289 | 15 | 0 |
| **LC47** | 84.6 | 36.2 | 7.3 | 297 | 22795 | 280 | 12 | 224 | 53 | 6 | 2 |
| **LC48** | 31.9 | 43 | 29.5 | 253 | 19353 | 293 | 14 | 182 | 52 | 4 | 1 |
| **LC49** | 62.2 | 18.9 | 2.3 | 727 | 55798 | 341 | 14 | 521 | 156 | 33 | 3 |
| **LC50** | 54.5 | 49.5 | 36.4 | 926 | 71071 | 193 | 220 | 640 | 57 | 8 | 1 |
| **LC patients median**  **(n=50)** | **36.3** | **36.6** | **11.9** | **928.5** | **71394.4** | **261.4** | **97.5** | **563.5** | **122.0** | **14.0** | **1.0** |

**Table S7:** Compiled data from HFAP (microclot) parameters, NETs markers and cirDNA in the long COVID (LC) patient cohort.

| **Figure 2** | **Category** | **p value** |
| --- | --- | --- |
| **A** | Total microclots | 1.97E-12 |
|  | 0-100 | <0.000000000000001 |
|  | 100-400 | 3.32E-12 |
|  | 400-900 | 2.03E-11 |
|  | 900-1600 | 2.36E-11 |
|  | >1600 | 5.33E-06 |
| **B** | Cirn-DNA | 2.01E-12 |
|  | MPO | 1.00E-15 |
|  | NE | 1.14E-09 |

**Table S8:** P value of the differences between Long COVID (LC) patients and healthy individuals (SA + EFS), in respect to microclot numbers (Figure 2A) and NETs markers and cirDNA (Figure 2B).

|  | | | | | | | |
| --- | --- | --- | --- | --- | --- | --- | --- |
|  | **CirDNA (ng/ml)** | **MPO (ng/ml)** | **NE (ng/ml)** | **Microclot**  **Objects/mL** | **Microclot count**  **in 0-100µm^2^ range** | **Microclot count**  **in 100-400µm^2^ range** | **Microclot count**  **in 400-900µm^2^ range** |
| Cir-nDNA (ng/ml) |  |  |  |  |  |  |  |
| MPO (ng/ml) | 0.186 |  |  |  |  |  |  |
| NE (ng/ml) | 0.086 | **3.38E-05** |  |  |  |  |  |
| Microclot Objects/mL | 0.941 | 0.763 | 0.324 |  |  |  |  |
| Microclot count in 0-100µm² range | 0.943 | 0.260 | 0.876 | **0.017** |  |  |  |
| Microclot count in 100-400µm² range | 0.979 | 0.899 | 0.232 | **0.00E+00** | **0.017** |  |  |
| Microclot count in 400-900µm² range | 0.832 | 0.238 | 0.453 | **1.14E-07** | 0.907 | **1.60E-06** |  |

**Table S9:** P values of the significance of the correlation between NETs markers, cirDNA and microclots in healthy individuals (SA + EFS). 0.00E+00 corresponds to Mann-Withney P-value with more than 15 decimals.

|  | | | | | | | |
| --- | --- | --- | --- | --- | --- | --- | --- |
|  | **CirDNA (ng/ml)** | **MPO (ng/ml)** | **NE**  **(ng/ml)** | **Microclot**  **Objects/mL** | **Microclot count in**  **0-100µm^2^ range** | **Microclot count in**  **100-400µm^2^ range** | **Microclot count in**  **400-900µm^2^ range** |
| Cir-nDNA (ng/ml) |  |  |  |  |  |  |  |
| MPO (ng/ml) | **0.017** |  |  |  |  |  |  |
| NE (ng/ml) | **0.009** | **6.65E-12** |  |  |  |  |  |
| HFAP Objects/mL | **0.002** | **0.001** | **0.001** |  |  |  |  |
| HFAP count in 0-100µm² range | **0.016** | **0.003** | **0.007** | **1.17E-11** |  |  |  |
| HFAP count in 100-400µm² range | **0.001** | **0.003** | **0.001** | **0.00E+00** | **2.26E-08** |  |  |
| HFAP count in 400-900µm² range | **0.015** | **0.016** | **0.007** | **0.00E+00** | **1.69E-04** | **0.00E+00** |  |

**Table S10:** P values of the significance of the correlation between NETs markers, cirDNA and microclots in patients with long COVID (LC). 0.00E+00 corresponds to Mann-Withney P-value with more than 15 decimals.

| **Size Range** | **Number of individuals with one or more microclots** | |
| --- | --- | --- |
|  | **Healthy Individuals (SA and EFS) (n=38)** | **Long COVID patients (LC, (n=50))** |
| **0-100 µm^2^** | 37 (97.3%) | 50 (100%) |
| **100-400 µm^2^** | 38 (100%) | 50 (100%) |
| **400-900 µm^2^** | 37 (97.3%) | 50 (100%) |
| **900-1600 µm^2^** | 26 (68.4%) | 49 (98%) |
| **>1600 µm^2^** | 5 (13.1%) | 30 (60%) |

**Table S11:** Number of individuals with one or more HFAP (microclots) in Long COVID (LC) patients and healthy individuals.
